# Supplementary material for: The global implications of a Russian gas pivot to Asia
Source: Nat Commun. 2025 Jan 4;16:386. doi: 10.1038/s41467-024-55697-7 (PMC11700154; doi:10.1038/s41467-024-55697-7)
Supplement: Supplementary file 1 — Supplementary Information [file 41467_2024_55697_MOESM1_ESM.pdf]

The global implications of a Russian gas pivot to Asia: Supplementary Information

Steve Pye[1,*], Michael Bradshaw[2], James Price[1], Dan Zhang[1], Caroline Kuzemko[3], Jack Sharples[4], Dan Welsby[5] & Paul E. Dodds[1,5]

[1] UCL Energy Institute, University College London, London, United Kingdom
[2] Warwick Business School, University of Warwick, Coventry, United Kingdom
[3] Department of Politics and International Studies, University of Warwick, Coventry, United Kingdom
[4] Oxford Institute for Energy Studies, Oxford, United Kingdom
[5] UCL Institute for Sustainable Resources, University College London, London, United Kingdom

* Corresponding author: s.pye@ucl.ac.uk

Contents

Contents ii
SI Section 1. Chinese gas demand uncertainty 1
SI Section 2. Additional modelling results for core scenarios 8
SI Section 3. Scenario sensitivity cases 11
SI 3.1. Russian gas exports 13
SI 3.2. European gas imports 15
SI 3.3. European gas supply 17
SI 3.4. Chinese gas supply 19
SI 3.5. Chinese gas imports 21
SI Section 4. Key assumptions in the TIAM-UCL Model 23
SI Section 5. Fossil fuel characterisation in TIAM-UCL 29
SI Section 6. Detailed representation of Supply curves for Russia and Saudi Arabia 43
References 47

SI Section 1. Chinese gas demand uncertainty

As the world's third-largest natural gas consumer behind the United States and Russia, China has rapidly transformed into a natural gas-buying behemoth in recent years to meet the growing demand for natural gas. China's natural gas consumption increase can be traced back to policies to address air pollution problems in coastal cities in 2000s, marked by the launch of the West–East Gas Pipeline in 2002[1]. In the subsequent decades, gas demand increased steadily driven by economic development and industrialization. Since 2015, when China submitted its initial Nationally Determined Contributions (NDCs), natural gas demand growth in China has been further accelerated by a series of government policies aimed at transitioning from coal to gas[2,3].

Even though the historical trend shows a rapid growth in demand for natural gas (See SI Figure 1), in the future the trend under emerging external and internal environment changes, including China's mitigation goals, gives rise to significant uncertainty. China's updated Nationally Determined Contributions (NDCs) in 2021 pledges to strictly limit the increase in coal consumption over the 14th Five-Year Plan (FYP) period (2021-2025) and phase it down in the 15th FYP (2026-2030), which potentially indicates a signal of sustained growth in natural gas demand[4]. However, China's gas consumption decreased by 1% in 2022 as a result of lower growth in economic and especially industrial activity, Covid-related restrictions and high prices[5]. In contrast, China's coal consumption grew by 4.6% in 2022 to a new all-time high of 4.5 billion metric tons[6]. Despite the recovery of natural gas demand in China in 2023, the government's policy stance on coal due to energy security concerns, along with a more rapid deployment of renewables driven by current climate commitments, has led to a high degree of uncertainty in the outlook for gas demand[7].

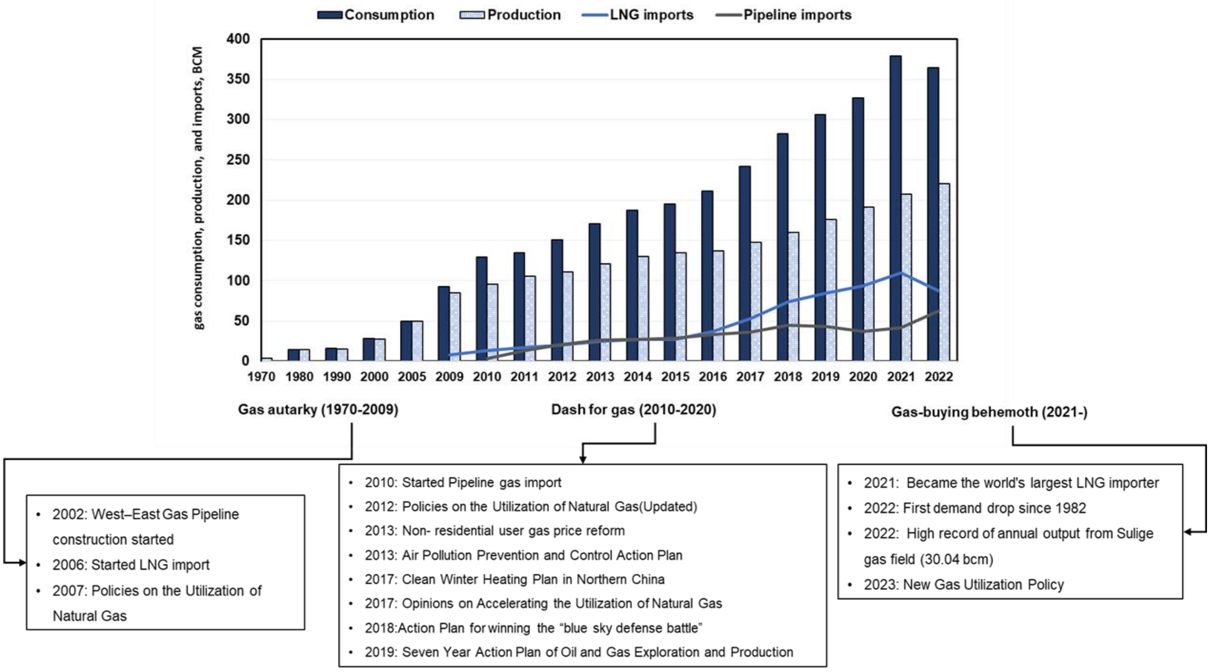

SI Figure 1. China gas consumption, production and imports from 1970-2021. Gas statistics used in this figure are sourced from IEA World Energy Balance.

Here we review existing research on China's gas demand, focusing on both domestic and international studies relating to China, using different research methods. Within these two scopes, three categories of literature are reviewed: i) China's gas demand forecast oriented studies, ii) China's low carbon scenarios studies, and iii) global scenarios modelling studies. To better compare the impacting factors of gas demand uncertainty, the identified characteristics for consideration in this review include:

- Projections approach: study focus on model demand change (in forecast studies) or assumptions applied to construct energy scenarios (in scenario studies).
- Geographical scope: global, multi-region, national or even provincial.
- Method (model) scope: narratives, econometrics, or energy system model – and whether energy system model is integrated or sectoral focus.
- Gas demand level: the demand volumes in the near term (2030) or long term (2050). It should be noted that, for some scenarios from commercial companies or institutes, demand levels are not published.

China-based national studies

Within China, research on future gas demand can be divided into two categories. One is gas demand forecast oriented, as SI Table 1 shows. These studies target forecasting near-term and temporal aggregated natural demand using historical time series data. This explains why these studies present a narrower range of 2030 gas demand (528-650 bcm) and typically do not provide a longer term outlook (2050). These studies exhibit notable strengths in more granularity of gas demand change both from spatial and temporal perspectives. For example, the engineering-based model constructed by Zhang et al.[8] represents the spatial-temporal variation of natural gas infrastructure at the provincial level to calculate the gas flow and GHG emissions from each province of China. However, it fails to capture changes in the broader energy system. The implications of various economic, technical, or social driving factors are embedded in time series analysis but are not explicitly discussed in terms of their respective impacts and emerging uncertainties (due to the approach taken).

SI Table 1. Summary of China's gas demand forecast oriented studies

Source	Key influencing factors	Geographical scope	Method (model) scope	Gas demand forecasting (BCM)	
				2030	2050
Ji et al., 2018[9]	Fuel prices, GDP	National and sectoral	Scenario analysis with econometric models	528	-
Li et al., 2020[10]	Energy supply side reform, industrialization and urbanization	National	The comprehensive energy elasticity coefficient method	650	-
National Energy Administration, 2021[11]	-	National	Annual review with historical data	550-600	-
Zhang et al., 2022[8]	Natural gas infrastructure, GHG emissions	Provincial	Scenario study-based inputs, network flow models, engineering-based models	576	500
Xu et al., 2023[12]	Seasonality	National	Econometric model (SARIMA model)	622	-

The other category of projections is from broader energy system perspective, which first identifies the factors impacting the energy system, such as economic development, policy instruments, then analyses these factors via scenarios, from which the gas demand outlook can be assessed. Since the announcement of the carbon neutrality target by President Xi in September 2020, numerous Chinese research institutes and scholars have assessed pathways for meeting national policy targets or different temperatures targets (1.5°C/2°C)[13,14]. Here, we mainly focus on those scenarios reporting different future gas demand, as shown in SI SI Table **2** shows. It is worth noting that the gas demand projected for 2050 in the results of Pan et al. (2020)[15] is significantly higher than in other studies. This difference arises because they did not include trade limitations and geopolitical barriers in their model, nor did they account for the early-arriving demographic tipping point of China.

These scenarios depict natural gas demand further into the future, and therefore show much greater uncertainty than 2030 studies, leading to substantial variability in demand across the ensemble. These variabilities arise partly due to different modelling methods and frameworks they employed. Additionally, data calibration of the base year in these scenarios is different, for example, the economical and demographical assumptions in research from various studies[15–17] are made pre-pandemic while CNPC's net zero road map includes more recent events like blackouts, strikes over fuel costs, and record high prices of generation fuels in 2021[18]. However, they all fail to capture the implications of broader geopolitical uncertainties, including China-US trade tensions, Russia-Ukraine war and so on.

SI Table 2. Summary of China's low carbon scenarios studies

Source	Scenarios (assumptions)	Geographical scope	Method (model) scope	Future gas demand, BCM	
				2030	2050
Pan et al., 2020[15]	NDC	Global	Global integrated assessment model (GCAM)	550	780
	NDC-HP (high oil price)			520	610
	NDC-LP (Low oil price			560	810
	2 ℃			550	710
	1.5 ℃			610	620
ICCSD, 2022[17]	Policy scenario	Global	Both bottom–up (BU) and top–down (TD) approaches. BU approaches include scenario analysis and sectoral focus models. TD approaches include a macro-model calculation and policy simulation.	518	654
	Reinforced policy scenario			579	503
	2 ℃			534	390
	1.5 ℃			496	210
SENR-RMU, 2021[16]	1.5 ℃	Global	PECE-LIU2020 model (LEAP framework) with additional modules of hydrogen and BECCS	600	352
CNPC, 2022[18]	Net Zero (2030 Carbon peak, 2060 Carbon net zero)	National	-	Gas peak at 650 bcm in 2040 then reduce to 410 bcm in 2060	

Global scenario modelling studies

Domestic analyses provide for a more granular and context-specific representation of the country's energy landscape and policy framework but may overlook the dynamic feedback of global climate policy, resulting gas trade and its impact on China's gas consumption. Global scenarios can provide such insights but do not provide the detailed representation of the Chinese energy system and economy. SI SI Table **3** presents some key global energy scenarios with estimates of China gas demand outlook in 2030 and 2050, including selected scenarios from IPCC AR6 ISO, international institutes, and oil and gas companies.

Scenarios from IPCC AR6 are categorised into C1 to C8 based on their climate policy ambition. Most of China's national low carbon scenarios target 1.5°C and 2°C-aligned climate policy, and / or NDC targets , (which global studies indicate could result in average global temperature increases of 2-3°C[19–21]. To reflect such policy ambition, C1-C6 scenarios from IPCC AR6 ISO database reporting China's gas demand are included for comparison. Most of these underlying scenarios are normative and based on the middle-of-the-road reference system (SSP2), using integrated assessment models (IAM)[22].

In these global scenario studies, variability in near-term gas demand is attributed to differences in the pace and scale of renewable energy deployment in the power sector, as well as the long-term roles of carbon capture, utilization, and storage (CCUS), and negative emissions technologies. The highest level of gas demand in 2050 is from Equinor's Wall scenario, where it assumes various barriers to global fossil fuel phase-out (e.g., limited capacity for wind and solar photovoltaic installation)[23]. In contrast, the IEA's STEPS scenario projects a strong rollout of renewables, which

reach nearly two-thirds of total power generation by 2050[24], compared to just over one-third in the IEEJ's reference scenario[25]. The Accelerated Scenario by BP, Advanced Technologies by IEEJ, and Bridge Scenario by Equinor assume more ambitious introduction and penetration of energy and environment technologies (e.g., CCS), accelerating the global energy transition[23,25,26]. Long-term gas demand outlooks differ across scenarios in account of the pace of industrial heat decarbonization, intermittent gas production in the power sector, and the role of natural gas as an input for hydrogen production.

In contrast to China-based national studies, the global studies see more conservative estimates for gas demand, in that most of them take account for some of the very latest global realities, such as the economic downturn after pandemic, and the implications of Ukraine crisis for gas trade. Apart from the differences in sectoral and technological assumptions, another key factor explaining the difference between IPCC and other global scenarios is the nature of scenarios, with most selected IPCC scenarios being normative, to describe pathways to a prespecified future while scenarios from IEA, Shell, and Equinor are exploratory, in that they define a set of starting conditions, such as policies and targets, and then see where they will lead. Even though global scenario modelling studies account for the impact of the global environment on China’s energy system, they lack detailed descriptions regarding China's recent economic downturn, and a shrinking population. In addition, faced with the current energy crisis, the representation of latest policy response from different governments and their implications are insufficient (such as RepowerEU, US Inflation Reduction Act).

SI Table 3. Summary of global scenario modelling studies

Source		Scenarios		Method (model) scope	Future gas demand, BCM	
					2030	2050
International agencies or institutes	IPCC, 2023[22]	1.5°C	C1 (n=69)	AIM/CGE, COFFEE, GCAM, GEM-E3_V2021, IMAGE, MESSAGEix-GLOBIOM, POLES ENGAGE, REMIND, REMIND-MAgPIE, WITCH 5.0	577	498
			C2 (n=93)		567	544
		2°C	C3 (n=256)		515	535
			C4 (n=122)		487	555
		For NDC's comparison	C5 (n=173)		479	713
			C6 (n=75)		502	784
	IEA, 2023[24]	Stated Policies Scenario (STEPS)		The Global Energy and Climate Model (GEC-M)	458	452
		Announced Pledges Scenario (APS)			410	185
	IEEJ, 2023[25]	Reference scenario		IEEJ Japan model: a regional model that combines an energy technology model based on MARKAL-JAPAN and an econometric, supply-demand analysis model.	392	488
		Advanced technologies scenario			376	295
	OPEC, 2022[27]	Reference case		OPEC World Energy Model (OWEM), World Oil Refining Logistics Demand (WORLD) model	458	574[1]
Oil and gas companies	BP, 2022[26]	Accelerated Scenario		-	471	275
		Net Zero Scenario			469	177
		New momentum Scenario[2]			481	592
	Shell, 2023[28]	Sky		Shell self-developed models: World Energy Model (WEM), Global Supply Model (GSM)	444	191
	Equinor, 2023[23]	Walls		-	546	622
		Bridges			465	147

[1] Due to the data availability in World oil outlook 2045 (OPEC,2022), this is the 2045 demand level.
[2] New Momentum is designed to capture the broad trajectory along which the global energy system is currently travelling.

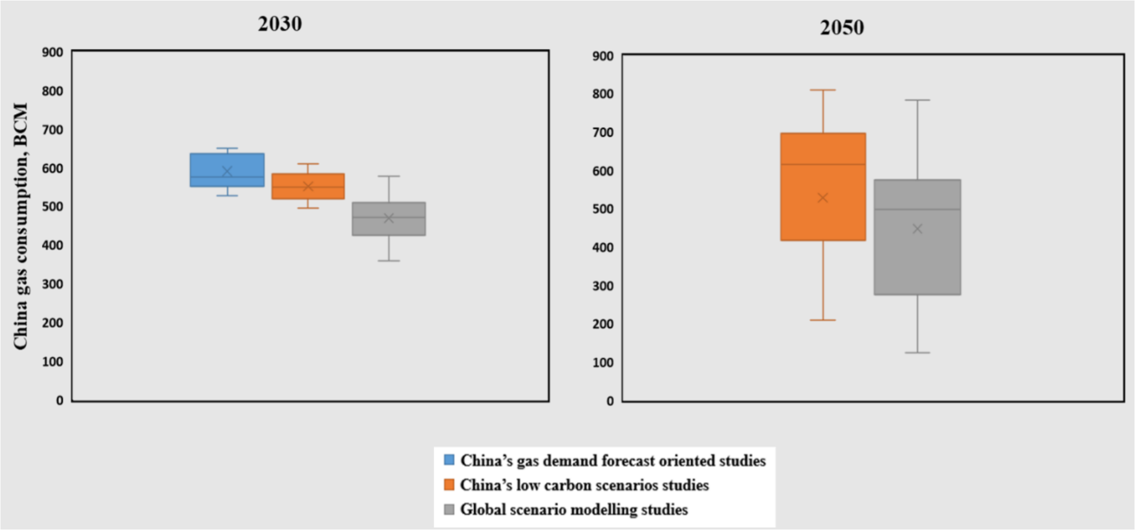

SI Figure 2. Ranges for China's projected gas demand in 2030 and 2050 by category of study. The box-and whisker plot shows the information provided in SI Tables 1-3. In the Box, the cross represents the average while the line represents the median.

To summarise, the ranges of China's gas demand for 2030 and 2050 for each category of study are presented in SI Figure 2.[3] It shows that the average values across studies are around 500-600 BCM in 2030, and there is a significant variance of long-term estimates in 2050, reflecting different paces of gas phase-out. Despite the abundant projections of China's gas demand in existing studies, there is a lack of understanding of the significant variability across scenarios. China's future economic performance and current concerns about national energy security open a key gap in current literature and raise the question of what will drive China's gas demand changes from now on.

[3] Note: Studies that do not run until 2050 are excluded in this figure, for example forecast oriented studies.

SI Section 2. Additional modelling results for core scenarios

This section includes additional model results from the core scenarios. Results for additional sensitivity cases are presented in SI section 3.

SI Table 4. Russia's gas exports by scenario. Results presented are sourced from the scenario modelling undertaken in this study using TIAM-UCL.

Note that cumulative losses for the period 2020 to 2050 for NDC cases, relative to REF, are 2127 (LM) and 1864 bcm (P2A). For B2D cases, these are 1589 (LM) and 1726 bcm (P2A).

	**NDC_REF**				**NDC_LM**				**NDC_P2A**			
	**2020**	**2030**	**2040**	**2050**	**2020**	**2030**	**2040**	**2050**	**2020**	**2030**	**2040**	**2050**
China	4	46	46	46	4	46	46	46	4	61	87	88
Europe	141	143	151	61	141	0	0	0	141	15	15	15
Turkey	19	0	0	7	19	15	11	7	19	0	11	7
LNG	40	22	47	83	40	52	83	121	40	64	63	84
Total	204	211	244	197	204	114	140	174	204	140	176	194
Change rel. to REF, Pipeline					0	-128	-140	-61	0	-114	-84	-4
Change rel. to REF, LNG					0	31	36	38	0	43	16	1
Change rel. to REF, Total					0	-97	-104	-23	0	-71	-68	-3
Change rel. to REF, Total (%)						-46%	-43%	-12%		-34%	-28%	-2%
Change rel. to 2020, Total (%)		4%	20%	-3%		-44%	-31%	-14%		-31%	-13%	-4%

	**B2D_REF**				**B2D_LM**				**B2D_P2A**			
	**2020**	**2030**	**2040**	**2050**	**2020**	**2030**	**2040**	**2050**	**2020**	**2030**	**2040**	**2050**
China	4	46	46	46	4	46	46	46	4	61	85	85
Europe	141	128	63	17	141	0	0	0	141	15	15	9
Turkey	19	0	0	0	19	6	0	0	19	0	0	0
LNG	40	22	20	27	40	52	61	39	40	52	27	0
Total	204	196	129	90	204	105	107	85	204	128	127	94
Change rel. to REF, Pipeline					0	-122	-63	-17	0	-98	-9	31
Change rel. to REF, LNG					0	31	41	12	0	31	6	-27
Change rel. to REF, Total					0	-91	-22	-5	0	-68	-2	3
Change rel. to REF, Total (%)						-47%	-17%	-5%		-35%	-2%	4%
Change rel. to 2020, Total (%)		-4%	-36%	-56%		-49%	-47%	-58%		-37%	-38%	-54%

SI Table 5. Europe's gas imports by scenario. Results presented are sourced from the scenario modelling undertaken in this study using TIAM-UCL.

Note that cumulative reductions for the period 2020 to 2050 for NDC cases, relative to REF, are 1773 (LM) and 2166 bcm (P2A). For B2D cases, these are 1284 (LM) and 1997 bcm (P2A).

	**NDC_REF**				**NDC_LM**				**NDC_P2A**			
	**2020**	**2030**	**2040**	**2050**	**2020**	**2030**	**2040**	**2050**	**2020**	**2030**	**2040**	**2050**
Africa	24	51	54	63	24	51	63	63	24	51	63	63
Azerbajan	8	9	19	19	8	22	22	22	8	22	22	22
Russia	141	143	151	61	141	0	0	0	141	15	15	15
LNG	73	14	17	29	73	85	60	48	73	98	67	41
Total	247	217	242	173	247	157	144	132	247	186	167	140
Change rel. to REF, Pipeline					0	-131	-140	-59	0	-116	-125	-44
Change rel. to REF, LNG					0	71	43	18	0	84	50	11
Change rel. to REF, Total					0	-60	-97	-40	0	-32	-75	-33

	**B2D_REF**				**B2D_LM**				**B2D_P2A**			
	**2020**	**2030**	**2040**	**2050**	**2020**	**2030**	**2040**	**2050**	**2020**	**2030**	**2040**	**2050**
Africa	24	51	63	46	24	51	63	47	24	51	63	46
Azerbajan	8	4	0	0	8	6	2	2	8	6	2	2
Russia	141	128	63	17	141	0	0	0	141	15	15	9
LNG	73	16	28	0	73	96	62	7	73	82	48	0
Total	247	199	153	63	247	153	127	56	247	154	128	57
Change rel. to REF, Pipeline					0	-126	-60	-14	0	-111	-45	-6
Change rel. to REF, LNG					0	80	34	7	0	66	20	0
Change rel. to REF, Total					0	-46	-26	-7	0	-44	-25	-6

SI Table 6. China's gas balance by scenario. Results presented are sourced from the scenario modelling undertaken in this study using TIAM-UCL.

	NDC_REF				NDC_LM				NDC_P2A			
	**2020**	**2030**	**2040**	**2050**	**2020**	**2030**	**2040**	**2050**	**2020**	**2030**	**2040**	**2050**
Production	176	184	250	299	176	184	252	303	176	184	263	299
Imports	125	161	221	232	125	161	223	232	125	161	233	265
Myanmar	*4*	*4*	*0*	*11*	*4*	*4*	*0*	*11*	*4*	*2*	*0*	*3*
Russia	*4*	*46*	*46*	*46*	*4*	*46*	*46*	*46*	*4*	*61*	*87*	*88*
Central-Asia	*25*	*56*	*81*	*81*	*25*	*44*	*81*	*81*	*25*	*44*	*81*	*81*
LNG	*92*	*54*	*93*	*93*	*92*	*66*	*95*	*93*	*92*	*54*	*64*	*93*
Total supply	300	345	471	530	300	345	476	535	300	345	496	563
% imports of supply	41%	47%	47%	44%	41%	47%	47%	43%	41%	47%	47%	47%

	B2D_REF				B2D_LM				B2D_P2A			
	**2020**	**2030**	**2040**	**2050**	**2020**	**2030**	**2040**	**2050**	**2020**	**2030**	**2040**	**2050**
Production	176	242	232	163	176	242	234	164	176	243	236	163
Imports	125	213	206	144	125	213	207	146	125	213	208	144
Myanmar	*4*	*5*	*11*	*11*	*4*	*8*	*11*	*11*	*4*	*5*	*5*	*11*
Russia	*4*	*46*	*46*	*46*	*4*	*46*	*46*	*46*	*4*	*61*	*85*	*85*
Central-Asia	*25*	*61*	*59*	*35*	*25*	*59*	*38*	*18*	*25*	*59*	*56*	*28*
LNG	*92*	*100*	*89*	*52*	*92*	*99*	*112*	*71*	*92*	*87*	*62*	*20*
Total supply	300	455	438	307	300	455	440	310	300	456	444	307
% imports of supply	41%	47%	47%	47%	41%	47%	47%	47%	41%	47%	47%	47%

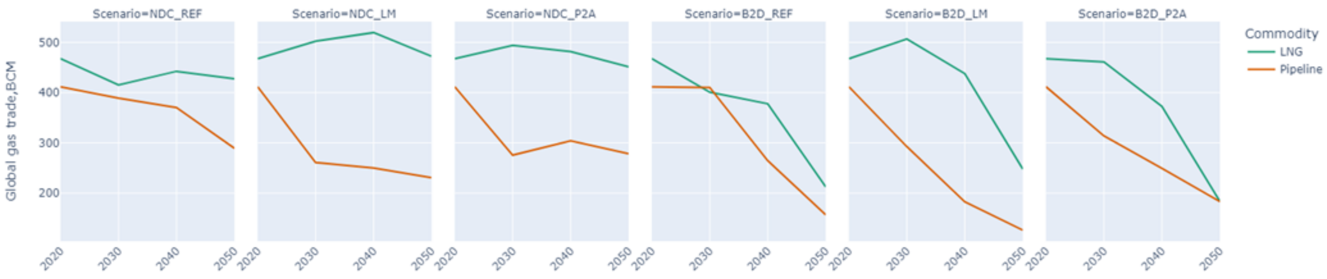

SI Figure 3. Global gas exports via LNG & pipelines by scenario. The results show trade flows between model regions, not intra-country pipeline flows. Results are sourced from the scenario modelling undertaken in this study using TIAM-UCL.

SI Section 3. Scenario sensitivity cases

To test key assumptions underpinning the core scenarios and their implications on the results, we developed a set of sensitivity cases. They particularly focus on the implications for Russian exports (both positive and negative) and the wider market. The three cases include -

1. **Reduced Chinese domestic production** (supply case): An important assumption in our current scenarios is that China will maintain the share of domestically produced gas as a percentage of total gas consumption. This is set at 50% based on current Chinese policy. Such an assumption influences the required level of imports so is important to explore. In this sensitivity case, we have relaxed this to a maximum production level to be 30%, which is aligned with the policy on domestic oil production share.

2. **Increased Chinese gas demand** (demand case): The demand for gas in our scenarios is, by design, largely driven by the climate policy ambition assumed. However, this does not necessarily capture the full range of possible gas demand levels in China, a key market which has implications for regional and global production. For this sensitivity, we have reviewed the range of gas demand levels in China (based on a review of the scenario literature, SI section 1) and implemented differentiated levels of demand in China that allow us to explore a broader range.

   We developed a case whereby we increased the amount of natural gas demand into the economy, primarily by relaxing constraints on uptake in different sectors. This enabled 2030 levels to reach 515-525 bcm in 2030 under B2D, a 15% increase compared to the previous level of 455 bcm, and 415-460 bcm in NDC, a 20-30% increase compared to the previous level of 345 bcm.

3. **Constraints on Russian LNG exports** (supply case): A key uncertainty concerns the ability of Russia to ramp up its LNG export business in the medium to long term, notably in LM where export routes via pipelines are somewhat constrained. Currently in LM we have assumed some level of near term constraint based on sanctions but with fewer restrictions in the medium to longer term. This includes LNG exports from Russia to the G7 and Europe being prohibited from 2030 in LM. This sensitivity brings more stringent restrictions on the ability of Russia to build out its LNG exports in the medium to long term. Our new medium to long term constraint on the ramp up of Russian LNG sees at most a 2% per year increase in export flows, placing much more friction on its growth.

   This is based on an outcome whereby insufficient ice-class carriers are supplied to enable full export from Yamal LNG and Arctic LNG due to western sanctions, compounded by EU sanctions on the transhipment of cargoes destined for non-EU markets. This is the status today, as outlined below.

   Russia has two large-scale LNG export terminals in operation: Sakhalin-II in the Far East and Yamal LNG in North-West Russia. Yamal LNG, based at Sabetta on the Yamal Peninsula, requires ice-class LNG carriers to load the cargoes, which are usually transferred to a regular LNG carrier elsewhere. The Yamal LNG project company has a transhipment agreement with Fluxys for transshipment at the Zeebrugge LNG terminal, and Novatek has an agreement with Elengy for transhipment at the Montoir-de-Bretagne LNG terminal in France.

Novatek’s latest project, Arctic LNG 2, began liquefaction operations in December 2023, but ceased in March 2024. The second train was towed to the site in July 2024. The project cannot launch, due to a lack of ice-class LNG carriers. 21 such carriers were ordered for the project from two shipyards in South Korea. The plan was for partial construction in South Korea, and finalisation at the Zvezda shipyard in Russia, with the participation for foreign companies. As it stands, five vessels were delivered to Zvezda, but it is unclear if they can be completed without foreign technology. In any case, those vessels have been made subject to western sanctions. The project companies for other proposed Russian LNG export projects, Murmansk LNG, Arctic LNG 1, and Arctic LNG 3, have all been added to the US sanctions list.

Novatek has placed two floating storage units (FSUs) at either end of the Norther Sea Route for transshipment that does not involve European LNG terminals, but those have also been sanctioned. The EU has now also sanctioned the transshipment of Russian LNG for onward delivery to non-EU markets. Even if Novatek is able to arrange the completion of a small number of ice-class LNG carriers, they will not be sufficient to enable Arctic LNG 2 to operate at full capacity. Even those that do operate will only be able to deliver to markets that do not fear western sanctions. If this situation persists, it will provide a substantial limitation to the build-out of Russia’s LNG export capacity.

The same metrics provided in the main paper are provided here, with plots showing the sensitivity case differences relative to the main scenarios. We have ordered the graphs are per the main manuscript, except present Chinese gas supply prior to Chinese gas imports.

SI 3.1. Russian gas exports

In all of the charts below, the sensitivity cases are compared to the core scenario, with absolute values for the core scenario (left hand panel) and relative change for sensitivity cases. For scenario / sensitivity case names, the first part of the scenario label denotes the climate policy ambition, NDC or B2D. The second part of the label denotes the geopolitical scenario, LM or P2A, or the counterfactual, REF. The third part of the label (only for sensitivity cases) refer to the following sensitivity cases –

1. LowChiGasProd - Reduced Chinese domestic production
2. HighChiGasCons - Increased Chinese gas demand
3. LowRusLNG - Constraints on Russian LNG exports

Note that the sensitivity case, LowRusLNG, is not included with the REF case as it only makes sense for the geopolitical cases, as it relates to limits resulting from sanctions. In the legend, 'Europe' refers to continental Europe, so excluding the UK.

We first consider Russian gas exports. Our working hypothesis is that cases 1 and 2 (increasing the need for Chinese imports) could see increased Russian exports, while case 3 (constraints on LNG export capacity) could decrease exports. In general, an increase in China's import demand does not significantly impact Russian exports in the two geopolitical cases, LM and P2A. This is largely because increased demand from China for LNG is being met by Australia, Middle East and North American producers (SI Figures 16-18). This analysis therefore suggests that even if Chinese demand was higher, Russian exports via LNG would not see strong growth, either to meet Chinese demand or LNG demand in other markets.

In case 3, LNG constraints have most impact on Russian LNG exports in NDC LM (SI Figure 5), reducing level by 40% in 2050, and to a lesser extent in NDC P2A (SI Figure 6), reducing the 2050 level by 14% in this sensitivity.

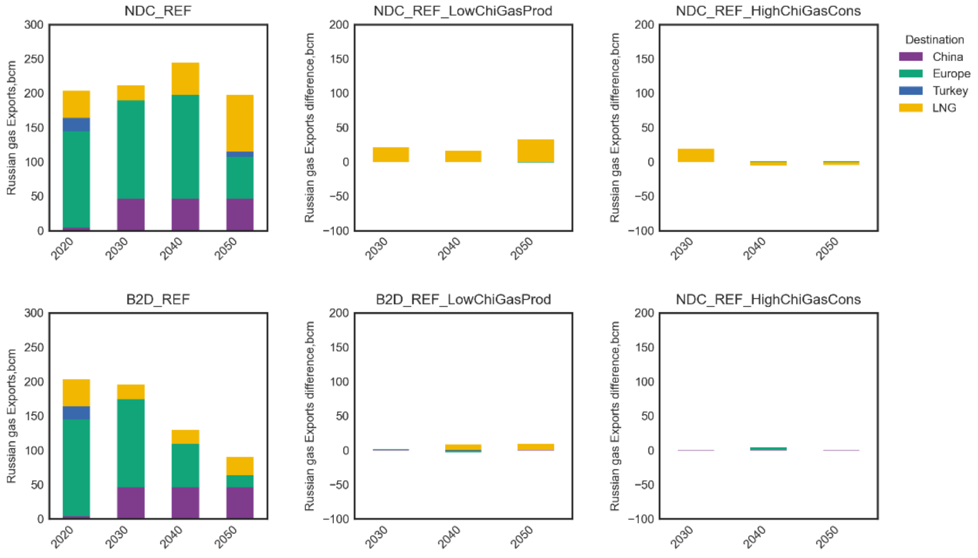

SI Figure 4. Russian gas exports - REF sensitivity cases. Regions specified represent pipeline export destination. Source: TIAM-UCL modelling

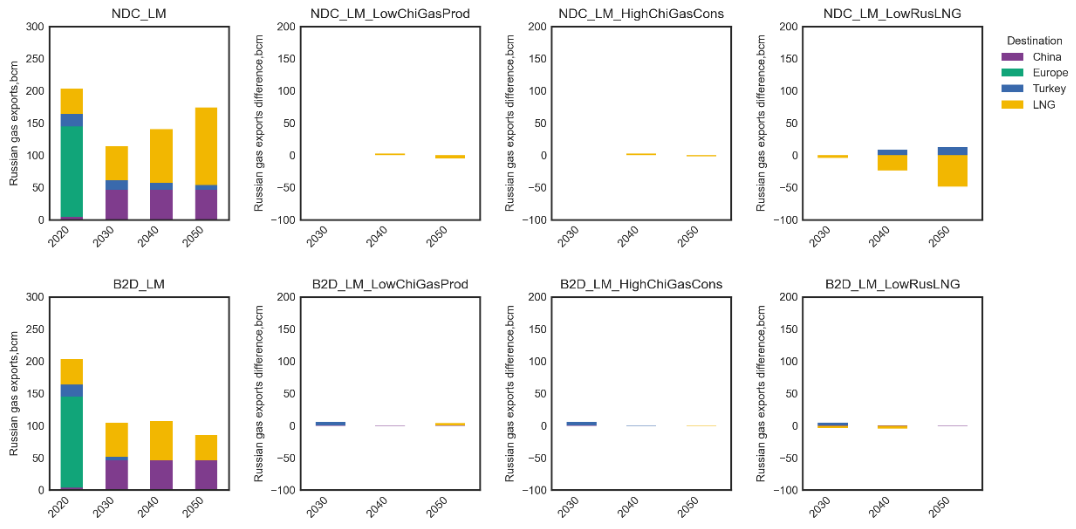

SI Figure 5. Russian gas exports – LM sensitivity cases. Regions specified represent pipeline export destination. Source: TIAM-UCL modelling

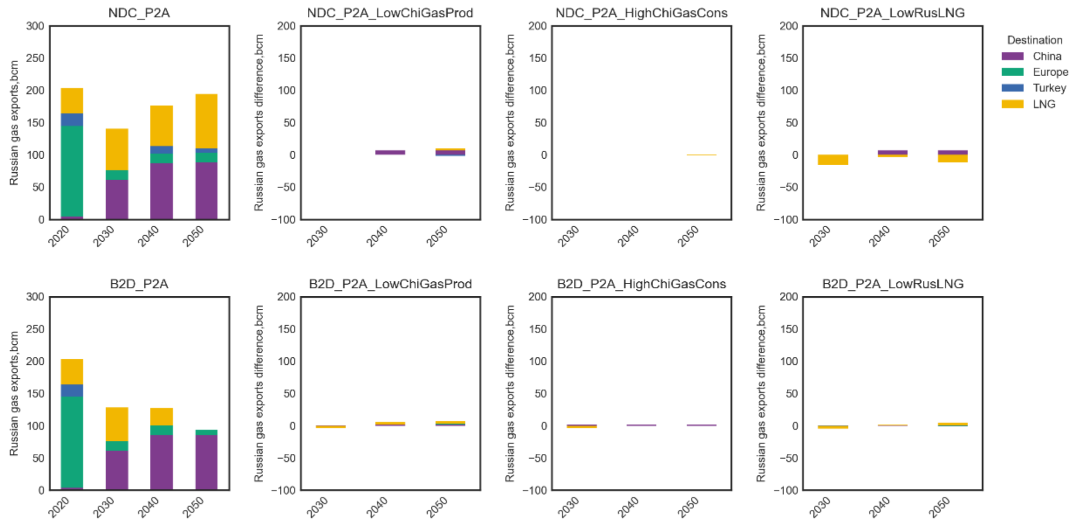

SI Figure 6. Russian gas exports – P2A sensitivity cases. Regions specified represent pipeline export destination. Source: TIAM-UCL modelling

SI 3.2. European gas imports

In all of the charts below, the sensitivity cases are compared to the core scenario, with absolute values for the core scenario (left hand panel) and relative change for sensitivity cases. For scenario / sensitivity case names, the first part of the scenario label denotes the climate policy ambition, NDC or B2D. The second part of the label denotes the geopolitical scenario, LM or P2A, or the counterfactual, REF. The third part of the label (only for sensitivity cases) refer to the following sensitivity cases –

1. LowChiGasProd - Reduced Chinese domestic production
2. HighChiGasCons - Increased Chinese gas demand
3. LowRusLNG - Constraints on Russian LNG exports

Note that the sensitivity case, LowRusLNG, is not included with the REF case as it only makes sense for the geopolitical cases, as it relates to limits resulting from sanctions.

In general, European demand for gas across the sensitivity cases is not strongly impacted in either LM or P2A. This reflects a general decline for imports in Europe over the model time horizon, particularly in the longer term. Some effects are seen in the short term in the NDC cases, in sensitivity cases 1 and 2, with decline in gas imports from Central Asia (Azerbaijan) which get diverted to China (2030), and a reduction in LNG (2040) (SI Figure 9). The LNG constraint on Russian exports has limited impact due to low dependence on this supply in future years in the core scenarios.

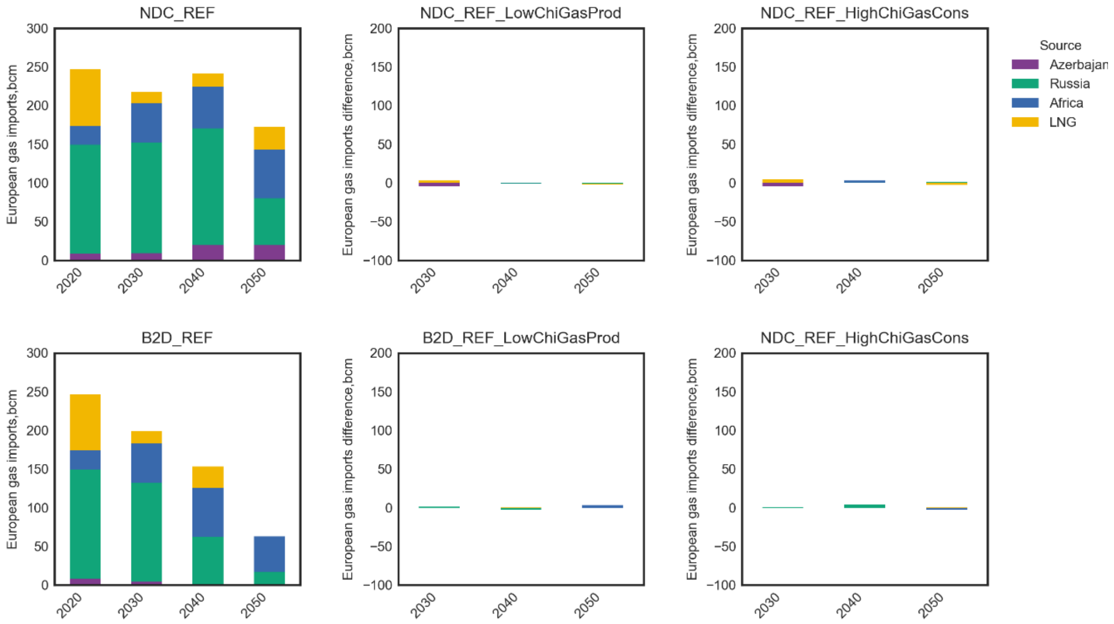

SI Figure 7. European gas imports - REF sensitivity cases. Regions specified represent the origin of pipeline imports to Europe. Source: TIAM-UCL modelling

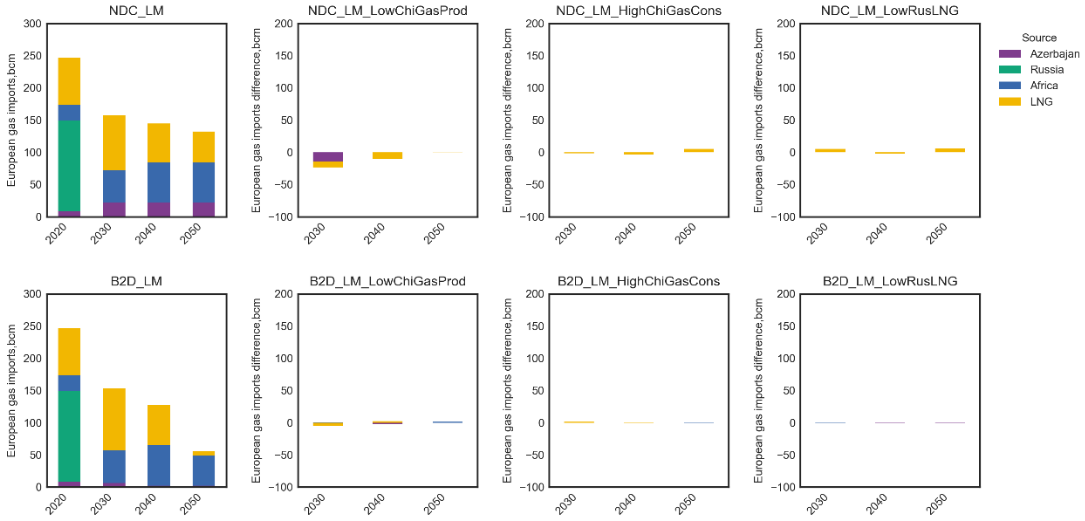

SI Figure 8. European gas imports – LM sensitivity cases. Regions specified represent the origin of pipeline imports to Europe. Source: TIAM-UCL modelling

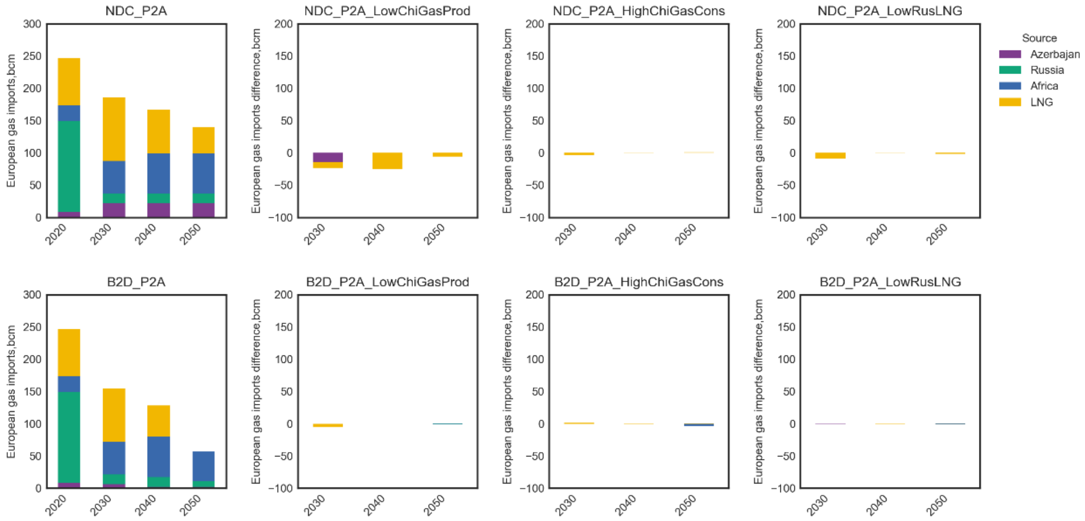

SI Figure 9. European gas imports – P2A sensitivity cases. Regions specified represent the origin of pipeline imports to Europe. Source: TIAM-UCL modelling

SI 3.3. European gas supply

In all of the charts below, the sensitivity cases are compared to the core scenario, with absolute values for the core scenario (left hand panel) and relative change for sensitivity cases. For scenario / sensitivity case names, the first part of the scenario label denotes the climate policy ambition, NDC or B2D. The second part of the label denotes the geopolitical scenario, LM or P2A, or the counterfactual, REF. The third part of the label (only for sensitivity cases) refer to the following sensitivity cases –

1. LowChiGasProd - Reduced Chinese domestic production
2. HighChiGasCons - Increased Chinese gas demand
3. LowRusLNG - Constraints on Russian LNG exports

Note that the sensitivity case, LowRusLNG, is not included with the REF case as it only makes sense for the geopolitical cases, as it relates to limits resulting from sanctions.

Given the limited impact on European gas imports across the sensitivity cases, there are limited changes observed for European gas supply. In those cases where imports decline, domestic production tends to meet the difference to maintain supply levels.

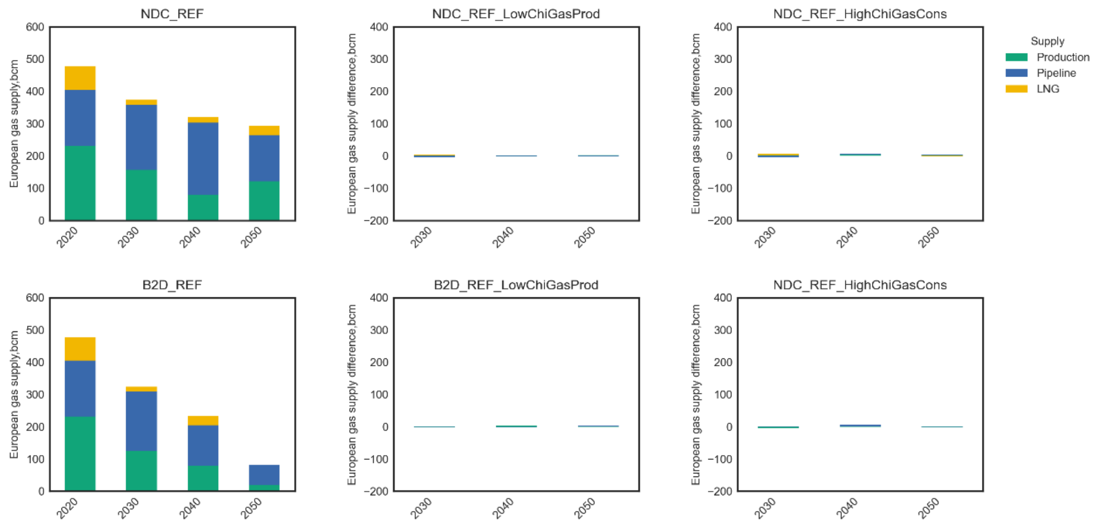

SI Figure 10. European gas supply - REF sensitivity cases. The data represent the origin of gas supply to Europe, with ‘production’ produced domestically, and imports via ‘pipeline’ and ‘LNG’. Source: TIAM-UCL modelling

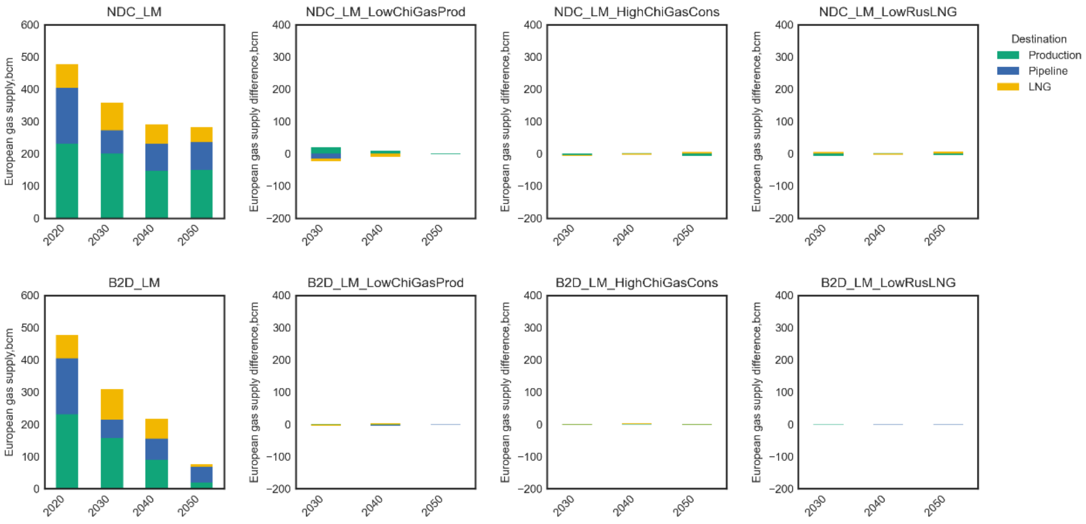

SI Figure 11. European gas supply – LM sensitivity cases. The data represent the origin of gas supply to Europe, with 'production' produced domestically, and imports via 'pipeline' and 'LNG'. Source: TIAM-UCL modelling

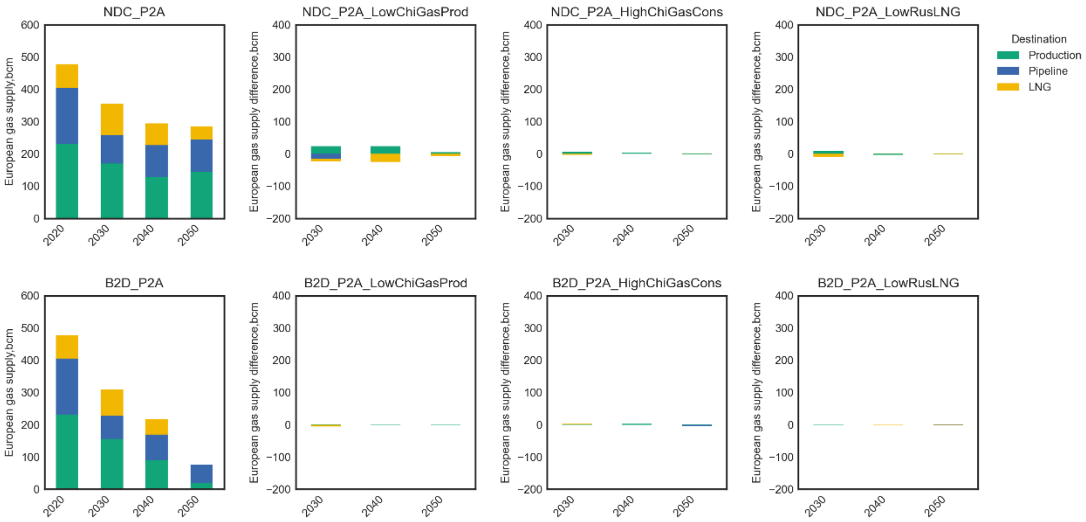

SI Figure 12. European gas supply – P2A sensitivity cases. The data represent the origin of gas supply to Europe, with 'production' produced domestically, and imports via 'pipeline' and 'LNG'. Source: TIAM-UCL modelling

SI 3.4. Chinese gas supply

In all of the charts below, the sensitivity cases are compared to the core scenario, with absolute values for the core scenario (left hand panel) and relative change for sensitivity cases. For scenario / sensitivity case names, the first part of the scenario label denotes the climate policy ambition, NDC or B2D. The second part of the label denotes the geopolitical scenario, LM or P2A, or the counterfactual, REF. The third part of the label (only for sensitivity cases) refer to the following sensitivity cases –

1. LowChiGasProd - Reduced Chinese domestic production
2. HighChiGasCons - Increased Chinese gas demand
3. LowRusLNG - Constraints on Russian LNG exports

Note that the sensitivity case, LowRusLNG, is not included with the REF case as it only makes sense for the geopolitical cases, as it relates to limits resulting from sanctions.

The implications of the sensitivity case 1 and 2, which increase gas imports to China due to lower domestic production (case 1) and higher demand (case 2), are shown below. In case 1, the change across both core scenarios, LM and P2A, is similar. Interestingly, LNG primarily compensates but does not completely make up for reduced domestic production, implying overall lower levels of gas supply. Case 2 sees a similar effect, with LNG compensating for domestic production declines, albeit at much lower levels due to the lesser impact of this sensitivity. Constraints on the build out of Russian LNG do not have a strong impact on China's gas supply, with most Chinese-bound cargoes coming from other producers.

These observations also hold for the import metrics provided in SI section 3.5.

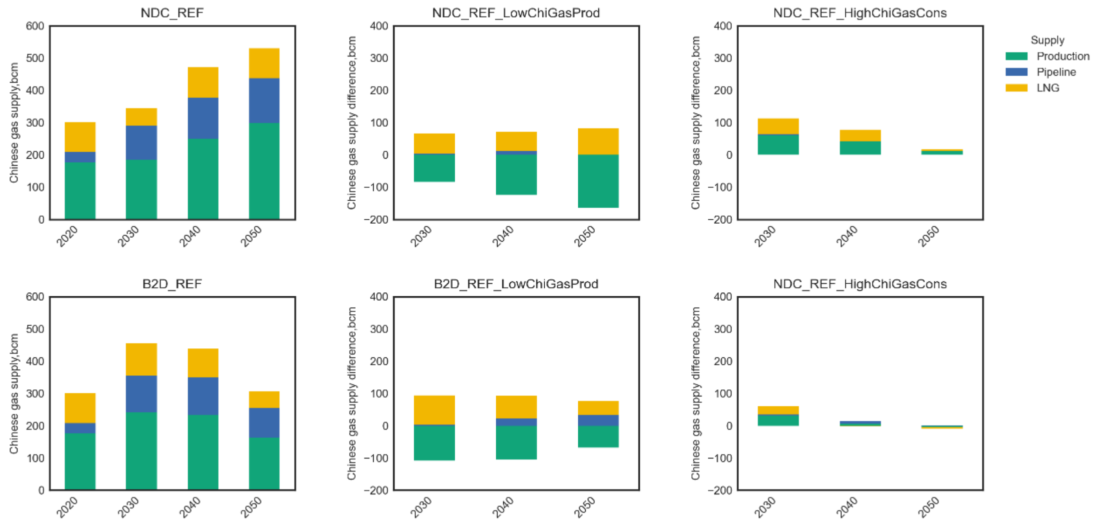

SI Figure 13. Chinese gas supply - REF sensitivity cases. The data represent the origin of gas supply to China, with 'production' produced domestically, and imports via 'pipeline' and 'LNG'. Source: TIAM-UCL modelling

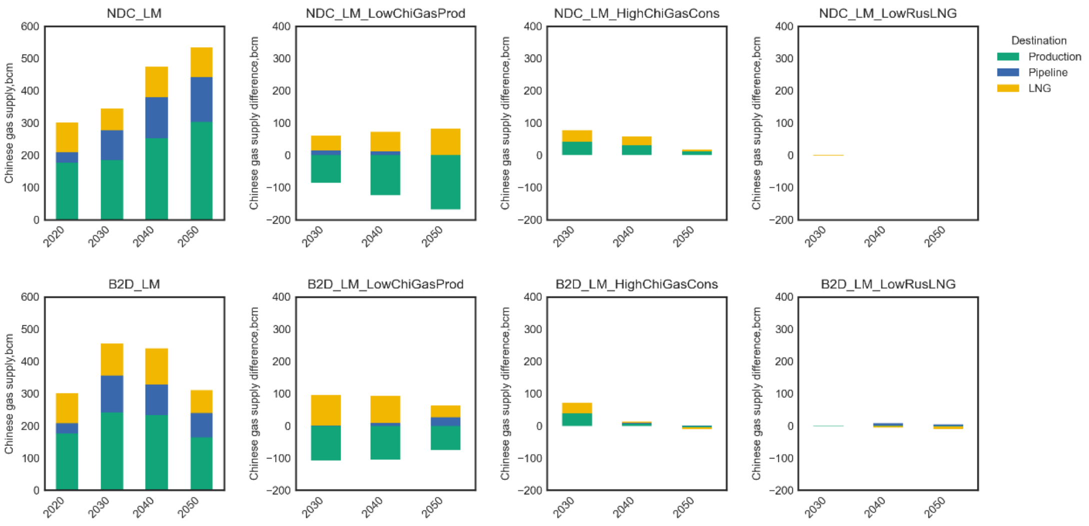

SI Figure 14. Chinese gas supply – LM sensitivity cases. The data represent the origin of gas supply to China, with ‘production’ produced domestically, and imports via ‘pipeline’ and ‘LNG’. Source: TIAM-UCL modelling

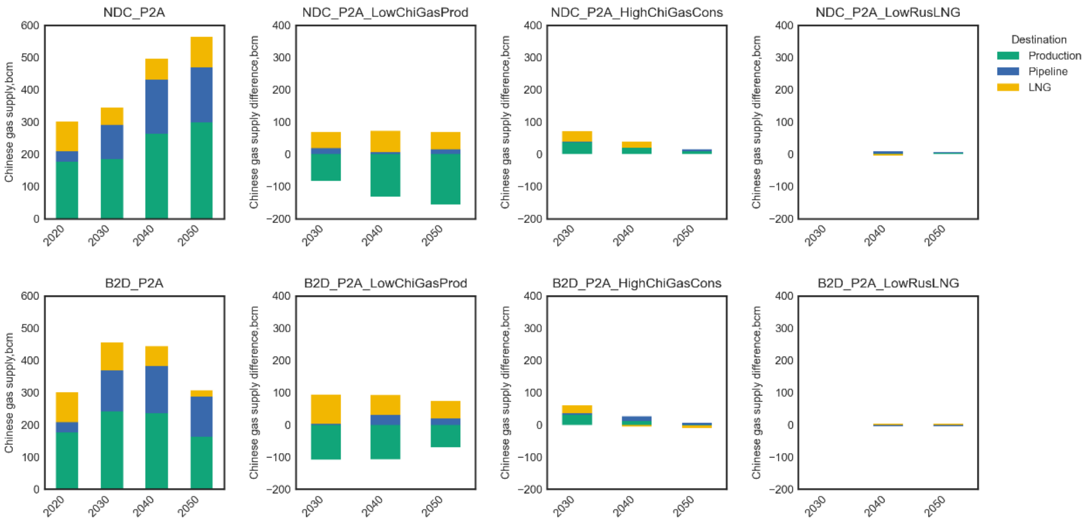

SI Figure 15. Chinese gas supply – P2A sensitivity cases. The data represent the origin of gas supply to China, with ‘production’ produced domestically, and imports via ‘pipeline’ and ‘LNG’. Source: TIAM-UCL modelling

SI 3.5. Chinese gas imports

In all of the charts below, the sensitivity cases are compared to the core scenario, with absolute values for the core scenario (left hand panel) and relative change for sensitivity cases. For scenario / sensitivity case names, the first part of the scenario label denotes the climate policy ambition, NDC or B2D. The second part of the label denotes the geopolitical scenario, LM or P2A, or the counterfactual, REF. The third part of the label (only for sensitivity cases) refer to the following sensitivity cases –

1. LowChiGasProd - Reduced Chinese domestic production
2. HighChiGasCons - Increased Chinese gas demand
3. LowRusLNG - Constraints on Russian LNG exports

Note that the sensitivity case, LowRusLNG, is not included with the REF case as it only makes sense for the geopolitical cases, as it relates to limits resulting from sanctions.

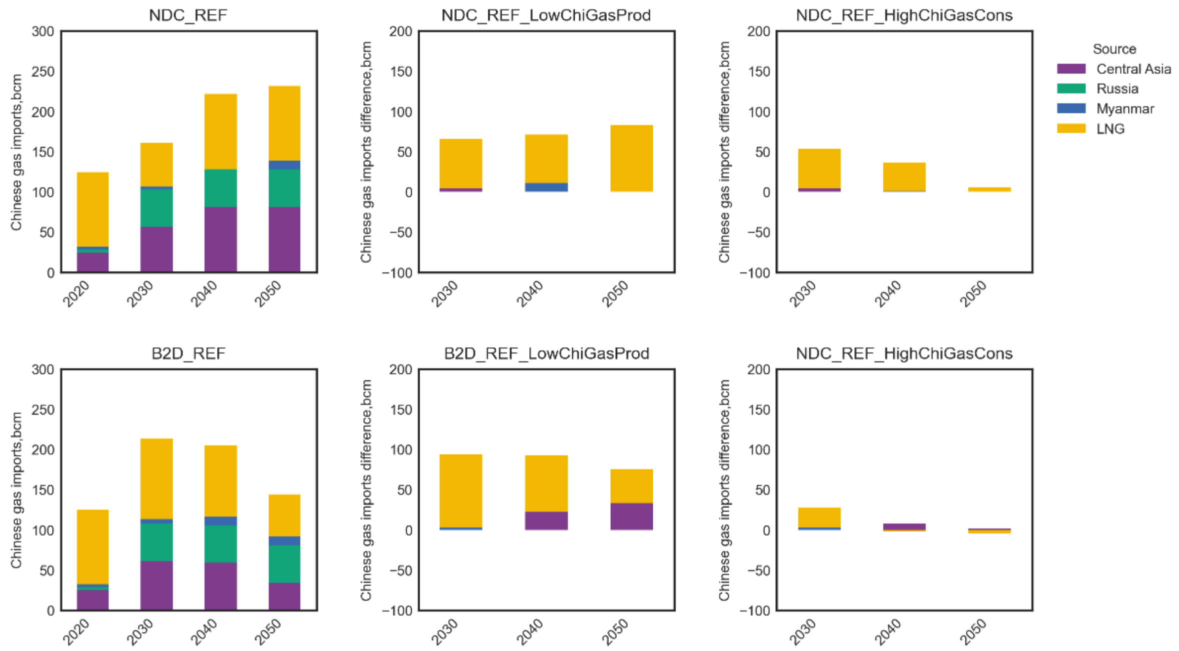

SI Figure 16. Chinese gas imports - REF sensitivity cases. Regions specified represent the origin of pipeline imports to China. Source: TIAM-UCL modelling

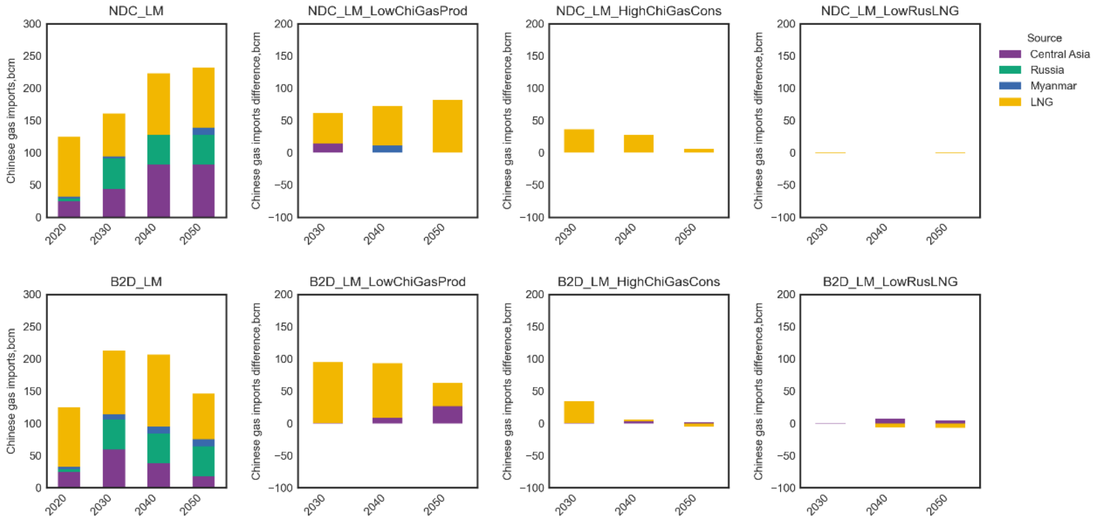

SI Figure 17. Chinese gas imports – LM sensitivity cases. Regions specified represent the origin of pipeline imports to China. Source: TIAM-UCL modelling

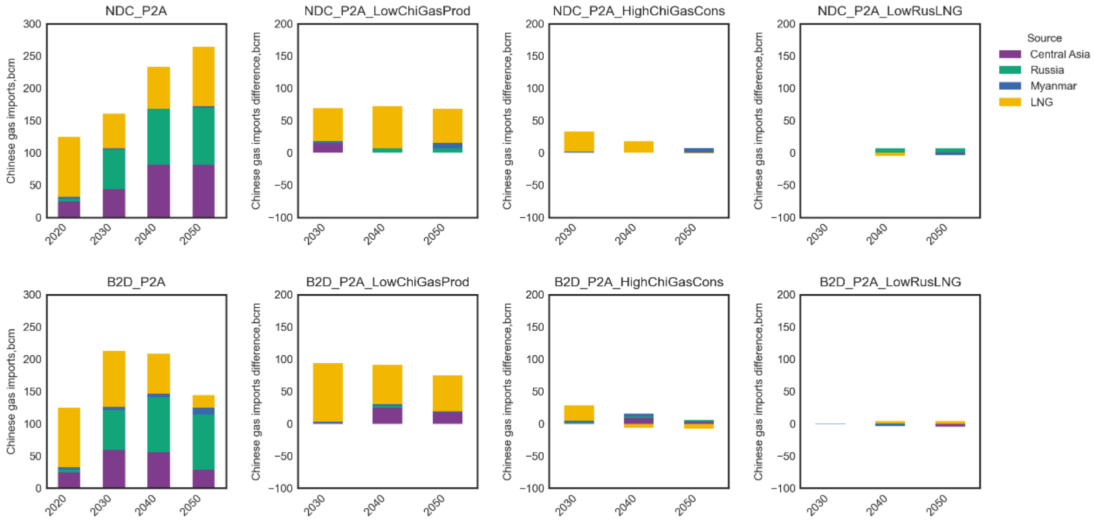

SI Figure 18. Chinese gas imports – P2A sensitivity cases. Regions specified represent the origin of pipeline imports to China. Source: TIAM-UCL modelling

SI Section 4. Key assumptions in the TIAM-UCL Model

This supplementary information describes some of the core assumptions used in this analysis. Additionally, the individual countries making up each region in TIAM-UCL are listed in SI Table 13.

Demand drivers

Population and economic growth drivers are based on the SSP2 'Middle of the Road' scenario narrative. These drivers are used to construct the energy service demands across different sectors. Some adjustments have been made to energy service demands to ensure final energy demand globally falls within the SSP2 marker model (MESSAGE) range. As SSPs are independent of climate ambition, defining the socio-economic backdrop that a given climate ambition has to be achieved within, the demands for SSP2 in TIAM have been tuned to match the marker model's base / reference SSP2 run with no climate constraints[29]. Region specific values are used but global values are shown in SI Table *7*.

SI Table 7. Demand drivers used in TIAM-UCL scenarios

Category	Assumption	Values				Units
		**2030**	**2050**	**2080**	**2100**	
SSP2	Population	8.3	9.2	9.4	9.0	billion
	GDP	17	25	42	59	000 US$2005/cap

Bioenergy characterisation and availability

Bioenergy is characterised into first and second generation fuels. First generation fuels are represented as bioliquids (bioethanol and biodiesel from crops which might compete with food crops for land) and biomethane (gas captured from controlled landfill sites). Four types of second-generation bioenergy feedstock distinguished: i) Solid biomass (BIOSLD), comprising woody residues from forestry and agriculture; ii) Energy crops (BIOCRP), comprising second generation purposely grown energy crops (grassy and woody bioenergy crops); iii) Municipal waste (BIOBMU), comprises wastes produced by households, industry, hospitals and the tertiary sector that are collected by local authorities; and iv) Industrial waste (BIOBIN), Solid and liquid products (e.g. tyres, sulphite lyes (black liquor), animal materials/wastes), usually combusted directly in specialised plants to produce heat and/or power. For each of these fractions cost supply curves are specified within the model for each of the 16 regions, i.e. amount of biomass available at different costs in each region. Cost ranges for solid biomass range between 4-16 $/GJ, and for energy crops between 9-15 $/GJ, with zero cost for waste fractions. To avoid competition for land, energy crops are assumed to be grown only on marginal and degraded land. Importantly, only solid biomass and energy crops fractions can be used for BECCS, and traded between regions. SI Table *8* lists the global potentials for each bioenergy type.

SI Table 8. Bioenergy availability (central estimates)

Bioenergy category	Values by year, EJ potential				Source
	2030	2050	2080	2100	
Solid biomass	43	45	48	50	[30]
Energy crops	17	31	31	31	Marginal land availability and energy crop yields from [31]. Biomass Feedstock Availability. Final report for BEIS. Supply cost curves based on [32]
MSW	17	27	27	28	TIAM-ETSAP

Conversion technology assumptions

A key input into the TIAM-UCL model is the assumptions on costs and performance of different conversion technologies, which produce low carbon vectors. This section provides an overview of the key assumptions for different technology groups. SI Table *9* provides information regarding the power generation sector. An important constraint for this sector is one that prevents unabated coal generation disappearing at too rapid a speed i.e. no faster than observed in the fastest power generation transition. For this, we use the phase out dates under the Powering Past Coal Alliance (PPCA)[33]. This is 2030 for OECD and EU members, and 2050 for others (except the European countries that have already committed to earlier dates).

The assumptions for the suite of BECCS technologies available in the model can be found in SI Table *10*. The main 'brake' on this technology set is the bioenergy resource availability. The other negative emission technology in TIAM-UCL is Direct Air Capture (DAC) which draws on heat and electricity inputs to capture CO2 directly from the atmosphere and sequester it. SI Table *11* has the cost and technical lifetime assumptions used for DAC.

On the supply side, hydrogen production technologies are divided into three different scales: centralised large-scale, centralised medium and decentralised small-scale production. Large-scale plants are based on biomass, coal and gas with continuous production of hydrogen. These plants are available with and without CCS technology. Hydrogen produced from centralised plants are transported with two different transportation options: long-distance pipe line transportation (gaseous hydrogen) and liquefaction plus trucks (liquid hydrogen). Hydrogen production data is presented in SI Table *12*, and are based on the review by Dodds and McDowall, 2012[34].

SI Table 9. Power generation costs and efficiency assumptions

	CAPEX, $2005 /kW					Efficiency, %			
**Technology**	**2010**	**2020**	**2030**	**2040**	**2050**	**2010**	**2020**	**2030**	**2050**
MSW combustion	5236	4862	4488		4114	23	27	30	33
Bioenergy combustion	2618	2431	2244		2057	28	31	34	37
Bioenergy combustion (dcn)	2880	2674	2468		2263	28	31	34	37
Bioenergy gasification	3080	2860	2640		2420	31	34	37	40
Bioenergy gasification (dcn)	3388	3146	2904		2662	31	34	37	40
Coal IGCC	2376					44	48	51	54
Coal super critical	1870					41	42	42	42
Coal ultra super critical	2277					46	48	49	50
Gas CCGT	990					56	59	61	63
Oil generation (dcn)	659					31	31	31	31
Oil generation	495					38	39	40	42
Coal IGCC w/CCS		3802	3564		3326		38	43	48
Coal USC w/CCS		3643	3416		3188		39	42	46
Gas CCGT w/CCS		1584	1485		1386		49	53	57
Geothermal shallow	2376	2310	2255	2200	2129				
Geothermal deep	3911	3644	3383	3108	2846				
Geothermal very deep		4978	4510	4015	3564				
Hydro dam	1650-6050	1623-5913	1595-5775	1568-5638	1540-5500				
Solar CSP	5850	3330	2700	2385	2070				
Solar PV	2587	667	437		288				
Tidal	6600	5500	4400		3432				
Offshore wind		3229	2507	1983	1591				
Onshore wind		1110	1011	949	919				
Nuclear Advanced LWR	4166	3939	3849		3623				
Storage	3300	1359	1051	700	510	80	80	80	80

Source: All costs presented above are averages across the 16 regions in TIAM-UCL, however in the model, costs are differentiated across the different regions. Fossil and CCS technologies (Ekins et al. (2017)[35]; Rubin et al. (2015)[36]); CCS is available from 2030, and can see capacity growth of a maximum of 5% per annum. Power generation technologies have capture rates of 90%, which do not improve over time. Future solar PV and wind reductions based on BNEF estimates (unpublished), recent cost estimates based on IRENA[37]. The maximum build rate of new solar PV and wind capacity each year is set at 30% of existing capacity in line with recent solar PV build rates[38]. Range for hydro denotes different resource tranches and cost of exploitation. In the above table, 'dcn' denotes 'decentralised'.

SI Table 10. a) BECCS technology costs and efficiency assumptions and b) other assumptions

a)

Technology Group	Technology Group	Efficiency %		Investment cost $/kW		Fix cost $/kW		Variable cost $/GJ	
	**Year**	**2030**	**2050**	**2030**	**2050**	**2030**	**2050**	**2030**	**2050**
Electricity	Energy Crop Combustion w CCS	26	31	3060	2618	175	131	6.9	6.6
	Energy Crop Gasification w CCS	29	34	3600	3080	206	173	1.7	1.7
	Solid Biomass Combustion w CCS	26	31	3060	2618	175	131	6.9	6.6
	Solid Biomass Gasification w CCS	29	34	3600	3080	206	154	1.7	1.7
Heat	Heat from biomass with CCS	63	65	1671	1419	189			
Hydrogen	Hydrogen from biomass gasification + CCS	42	44	4594	3516	322	246		
Advanced transport fuels	FT process w CCS using solid biomass	34	42	2235	1565	27	27	0.8	0.8
	FT process w CCS using energy crops	34	42	2235	1565	27	27	0.8	0.8

b)

Technology Group	Technology Group	Start time	Life yr	Availability / capacity factor	CO_2 Capture rate %	Build rate %
Electricity	Energy Crop Combustion w CCS	2030	25	0.85	90	5
	Energy Crop Gasification w CCS					
	Solid Biomass Combustion w CCS					
	Solid Biomass Gasification w CCS					
Heat	Heat from biomass with CCS	2030	30	0.6	90	3
Hydrogen	Hydrogen from biomass gasification + CCS	2030	30	0.9	90	5
Advanced transport fuels	FT process w CCS using solid biomass	2030	30	0.9	50	5
	FT process w CCS using energy crops					

Source: Butnar et al. 2020[39]

SI Table 11. Direct Air Capture costs and technical lifetime assumptions[40]

Technology	Life (yr)	Fixed O&M costs ($2005/tCO2/yr)	Capital investment costs ($2005/t$CO_2$)
		2030	2030
Direct air capture	20	120	2900

SI Table 12. Hydrogen production technology costs and efficiency assumptions

Technology	Size	Fixed O&M costs (% capital costs)	Capital investment costs ($2005/GJ/y)	
			2025	2050
Coal gasification	Large	0.05	27	24
Gas SMR	Large	0.04	7	5
Gas SMR	Small / medium	0.04	17	14
Biomass gasification	Large	0.07	26	26
Biomass gasification	Medium	0.07	34	34
Biomass gasification	Small	0.07	43	43
Waste gasification	Medium	0.07	34	34
Electrolysis	Large	0.04	7.7	2.4
Electrolysis	Medium	0.05	8.8	2.7
Electrolysis	Small	0.05	10	3.1

SI Table 13. List of regions and countries in the 16 region TIAM-UCL model

Region	Countries
Africa (AFR)	Algeria, Angola, Benin, Botswana, Burkina Faso, Burundi, Cameroon, Cape Verde, Central African Republic, Chad, Comoros, Congo, Côte d'Ivoire, Democratic Republic of the Congo, Djibouti, Egypt, Equatorial Guinea, Eritrea, Ethiopia, Gabon, Gambia, Ghana, Guinea, Guinea-Bissau, Kenya, Lesotho, Liberia, Libya, Madagascar, Malawi, Mali, Mauritania, Morocco, Mozambique, Namibia, Niger, Nigeria, Rwanda, Sao Tome and Principe, Senegal, Seychelles, Sierra Leone, Somalia, South Africa, South Sudan, Sudan, Swaziland, Togo, Tunisia, Uganda, United Republic of Tanzania, Zambia, Zimbabwe
Australia (AUS)	Australia, New Zealand
Canada (CAN)	Canada
Central and South America (CSA)	Anguilla, Antigua and Barbuda, Argentina, Aruba, Bahamas, Barbados, Belize, Bermuda, Bolivia, Brazil, Cayman Islands, Chile, Colombia, Costa Rica, Cuba, Dominica, Dominican Republic, Ecuador, El Salvador, Falkland Islands, Grenada, Guatemala, Guyana, Haiti, Honduras, Jamaica, Martinique, Netherlands Antilles, Nicaragua, Panama, Paraguay, Peru, Saint Kitts and Nevis, Saint Lucia, Saint Vincent and the Grenadines, Suriname, Trinidad and Tobago, Uruguay, Venezuela (Bolivarian Republic of)
China (CHI)	China, Taiwan, Tibet
Eastern Europe (EEU)	Bosnia and Herzegovina, Bulgaria, Croatia, Czech Republic, Hungary, Montenegro, Poland, Romania, Serbia, Slovakia, Slovenia, The former Yugoslav Republic of Macedonia
Former Soviet Union (FSU)	Armenia, Azerbaijan, Belarus, Estonia, Georgia, Kazakhstan, Kyrgyzstan, Latvia, Lithuania, Republic of Moldova, Russian Federation, Tajikistan, Turkmenistan, Ukraine, Uzbekistan
India (IND)	India
Japan (JAP)	Japan
Mexico (MEX)	Mexico
Middle-east (MEA)	Bahrain, Brunei Darussalam, Cyprus, Iran (Islamic Republic of), Israel, Jordan, Kuwait, Lebanon, Occupied Palestinian Territory, Oman, Qatar, Saudi Arabia, Syrian Arab Republic, Turkey, United Arab Emirates, Yemen
Other Developing Asia (ODA)	Afghanistan, American Samoa, Bangladesh, Bhutan, Cambodia, Democratic People's Republic of Korea, Fiji, French Polynesia, Indonesia, Kiribati, Lao People's Democratic Republic, Malaysia, Maldives, Mauritius, Mongolia, Myanmar, Nepal, New Caledonia, Pakistan, Papua New Guinea, Philippines, Samoa, Singapore, Solomon Islands, Sri Lanka, Thailand, Timor-Leste, Tonga, Vanuatu, Vietnam
South Korea (SKO)	Republic of Korea
United Kingdom (UK)	United Kingdom
USA (USA)	United States of America
Western Europe (WEU)	Albania, Andorra, Austria, Belgium, Denmark, Faroe Islands, Finland, France, Germany, Gibraltar, Greece, Greenland, Iceland, Ireland, Italy, Luxembourg, Malta, Monaco, Netherlands, Norway, Portugal, San Marino, Spain, Sweden, Switzerland, Vatican

SI Section 5. Fossil fuel characterisation in TIAM-UCL

This section briefly discusses the upstream representation of fossil fuels in TIAM-UCL. This includes the cost and availability of different categories of fossil fuels, and asymmetric constraints which constrain how quickly production can grow or decline.

Coal

Primary coal resources in TIAM-UCL are split into two categories, utilizing data collected by Remme et. al.[41]:

- Brown coal (lignite): lower energy content, with average heating value ranging from 5.57-17 MJ/kg
- Hard coal (sub-bituminous, bituminous and anthracite): higher energy content, with average heating value ranging from 17.58-27.55 MJ/kg

As with oil and fossil methane gas, the extraction technologies for coal split the resource base into cost tranches, in order to reflect (albeit simplistically), cost depletion dynamics. In short, as the more accessible and higher quality resources are depleted, the model must move to more expensive extraction of (potentially) harder to exploit resources. Coal is split into hard coal and brown coal, with the representative mining technologies for both categories split into three cost categories. The distribution of resources/reserves assigned to each cost category varies by region, and is influenced by the proportion of the total resource base which can be considered reserves, with the remainder of resources split between the middle and highest cost categories.

SI Figure 19 shows a cost depletion curve for global coal resources, and the corresponding global supply cost curve constructed from the cost depletion curve. Additionally, SI Figure 20 shows a global supply cost curve broken down into the regions of TIAM-UCL.

a)

b)

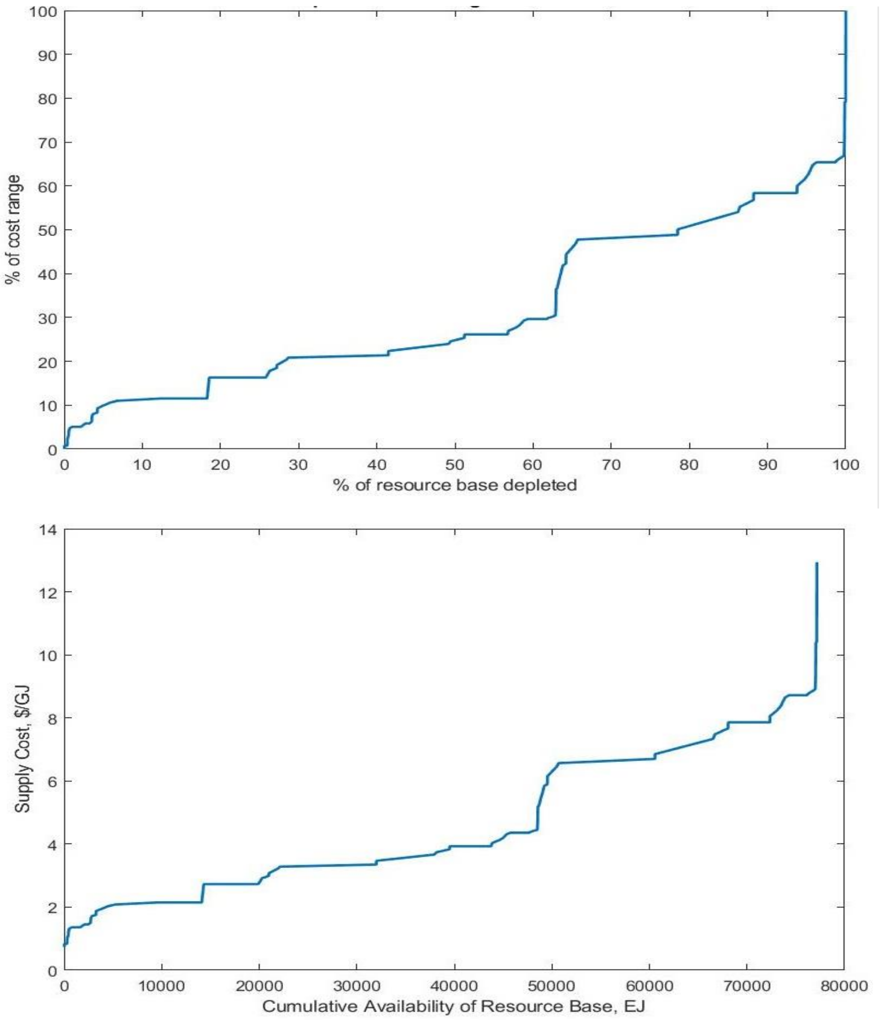

SI Figure 19. a) Cost depletion curve derived from TIAM-UCL resources and costs for global coal; b) supply cost curve derived from the cost depletion curve. a) Depletion curve shows how costs increase as the overall resource base is depleted, while b) provides the cost supply curve derived from a), based on the actual resource levels. Republished from Welsby, D., Price, J., Pye, S. & Ekins, P. Unextractable fossil fuels in a 1.5 °C world. Nature 597, 230–234 (2021)[42] under Copyright © 2021, The Author(s), under exclusive licence to Springer Nature Limited.

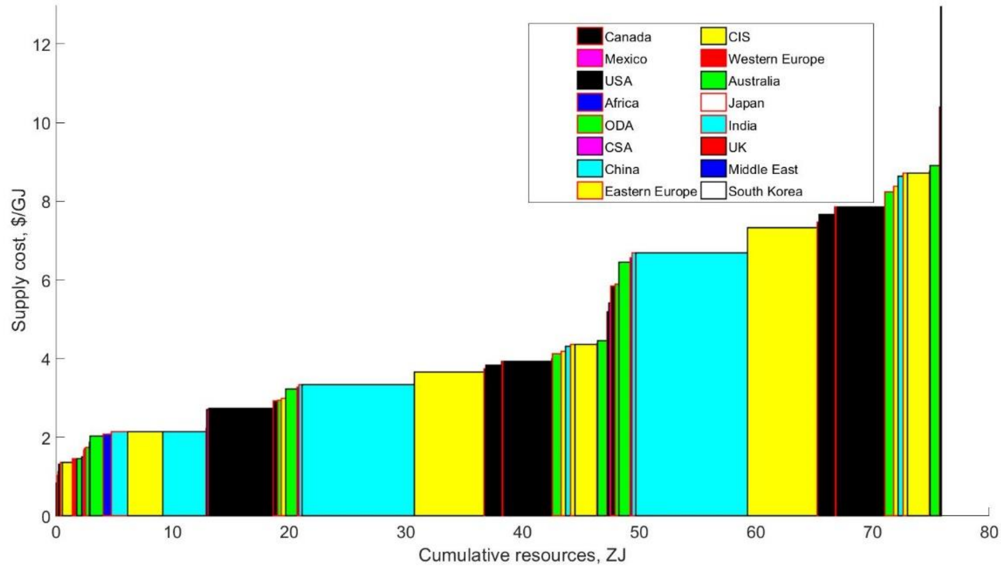

SI Figure 20. Global supply cost curve for coal disaggregated into TIAM-UCL regions. Supply cost curve disaggregating SI Figure 19 into TIAM-UCL modelled regions. Republished from Welsby, D., Price, J., Pye, S. & Ekins, P. Unextractable fossil fuels in a 1.5 °C world. Nature 597, 230–234 (2021)[42] under Copyright © 2021, The Author(s), under exclusive licence to Springer Nature Limited.

<u>Fossil methane gas</u>

The underlying availability and cost of fossil methane gas in TIAM-UCL is disaggregated into the following geological categories:

- Non-associated conventional gas proved reserves
- Non-associated conventional gas reserve additions
- Non-associated conventional gas new discoveries
- Associated fossil methane gas
- Arctic conventional fossil methane gas resources
- Shale gas
- Coal bed methane
- Tight fossil methane gas

As with oil, the disaggregation of fossil methane gas in TIAM-UCL is based on McGlade[43]. This analysis has been extended in Welsby[44], with field-level assessments of resource availabilities and costs. On a regional level, supply cost curves are constructed using an approach developed in Welsby[44]. Resource assessments were generally conducted at disaggregated field-/play-level, and then aggregated into the regions of TIAM-UCL using probability distributions, and taking into account any correlation between discrete estimates etc. These were then applied to depletion curves which were formed from a database of field-/play-level costs where possible. The database was extended to fields for which costs were either not known (i.e. no publicly available indication of field supply costs) or have not yet been developed.

SI Figure 21 shows an example cost depletion curve for conventional non-associated fossil methane gas reserves (taken from Welsby[44]). Aggregated regional supply cost curves (SI Figure 22) were derived from field-/play-level cost depletion curves shown in SI Figure 21 and have been aggregated into the TIAM-UCL regions. The three-step cost curves in TIAM-UCL mean that costs for each regions are aggregated from field-level data into weighted average costs for each cost step.

a)

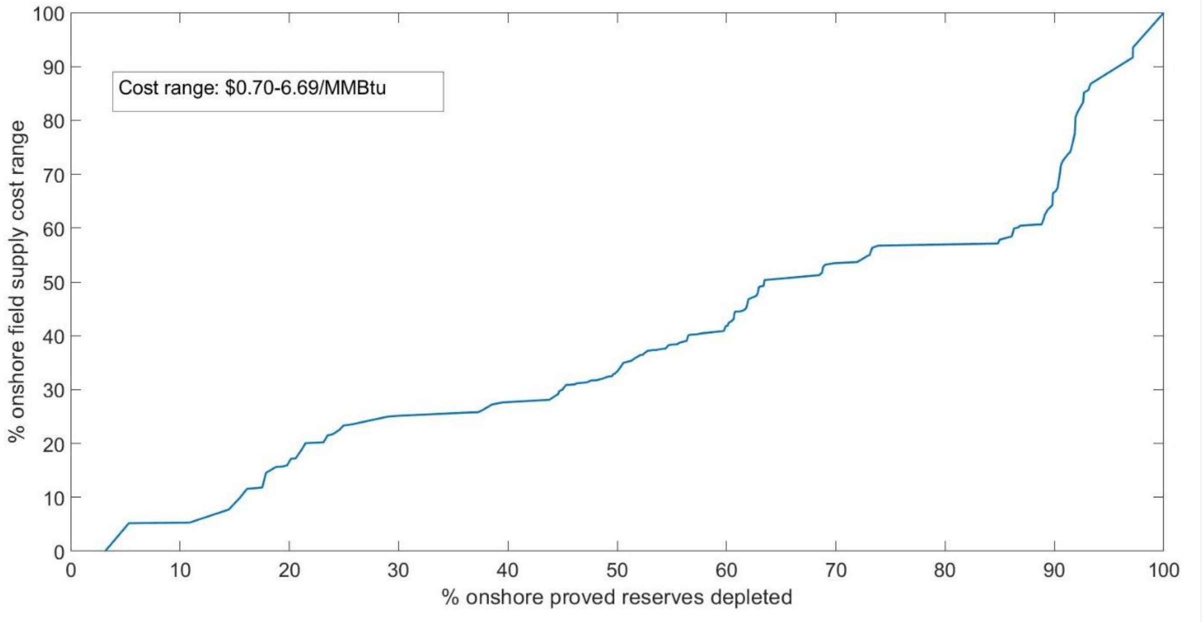

b)

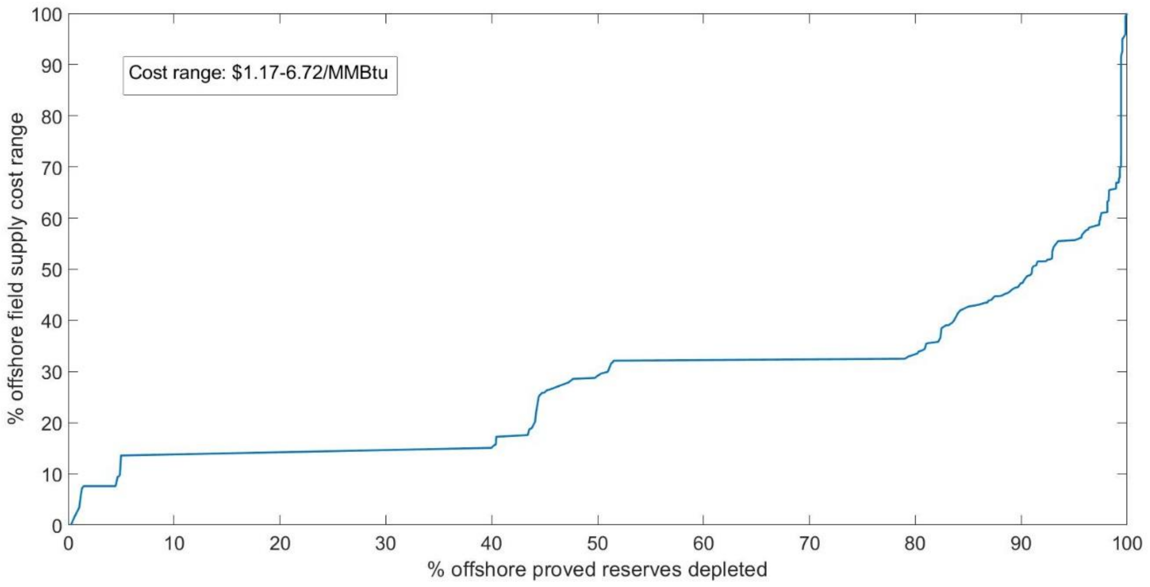

SI Figure 21. Example cost depletion curve for proved onshore (a) and offshore (b) non-associated gas reserves. Depletion curve shows how costs increase as the overall resource base is depleted. Republished from Welsby, D. Modelling uncertainty in global gas resources and markets. (University College London, London, 2022)[44], and released under a Creative Commons Attribution-Non Commercial 4.0 International Licence.

Additionally, SI Figure 22 shows a) the regional breakdown of the resource distribution, and b) the supply cost with each resource category identified. For reference, none of the figures in this section include associated fossil methane gas resources in the supply cost curves, as these are calculated separately, with resource availabilities calculated by McGlade[43] and Welsby[44]. Additionally, improvements made by Welsby[44] include an endogenous decision within the model of whether to produce the gas (which requires investment in new capacity if existing capacity is insufficient) or flare/vent it.

a)

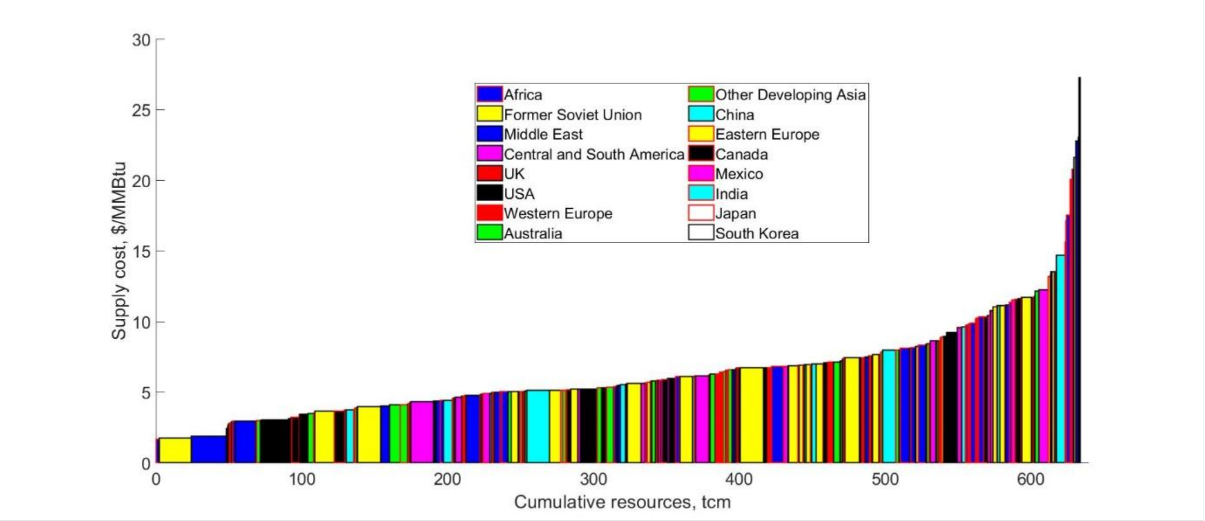

b)

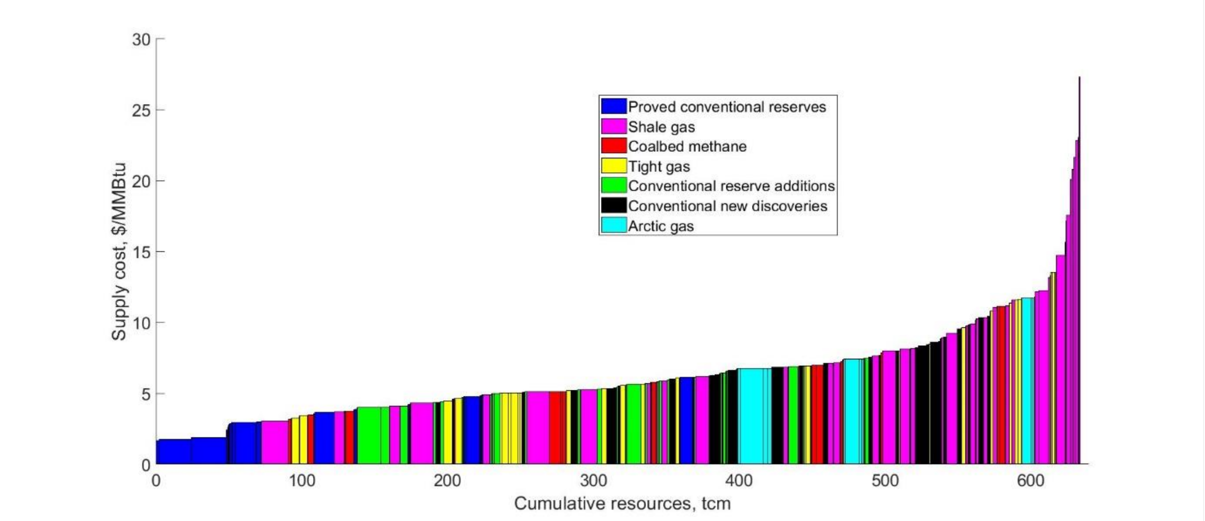

SI Figure 22. Global gas supply cost curve from 2015. a) disaggregates supply curve by region and b) by resource category. Republished from Welsby, D., Price, J., Pye, S. & Ekins, P. Unextractable fossil fuels in a 1.5 °C world. Nature 597, 230–234 (2021)[42] under Copyright © 2021, The Author(s), under exclusive licence to Springer Nature Limited.

SI Table 14 shows a cost range for some key fossil methane gas mining technologies, generated using a field-level database and a linear regression model applied to geological parameters to generate cost depletion curves[44].

SI Table 14. Cost range for individual fossil methane gas fields derived. Based on regression analysis by Welsby[44], and used to construct supply cost curves implemented in TIAM-UCL

Resource category	Minimum cost, $/boe	Minimum cost region	Maximum cost, $/boe*	Maximum cost region
Proved non-associated onshore conventional reserves (includes sour)	4	FSU	38	FSU
Proved non-associated offshore shallow conventional reserves	7	Middle East	37	Europe
Proved non-associated offshore deep conventional reserves	16	Central and South America	38	USA
Conventional non-associated reserve additions	10	FSU	45	Middle East
Undiscovered non-associated conventional	24	Middle East	58	Central and South America
Sour fossil methane gas undeveloped	29	FSU	45	MEA_P
Arctic (undeveloped)	36	-	63	-
Shale gas	14	USA	147	MEA_P
Tight fossil methane gas	18	USA/CAN	66	Europe
CBM	17	USA	60	China, FSU

* Fossil methane gas costs here have been expressed in $/boe so they can be directly compared to the oil extraction costs in Table 1.3. Data from Welsby, 2022[44], McGlade, 2013[43].

Oil

The representation of oil in TIAM-UCL is predominantly based on the work by McGlade[43], which focused on quantifying uncertainties in the outlook for oil and fossil methane gas, and in particular their availability and costs. As with fossil methane gas, oil is split into different geological categories, each with specific availabilities and supply cost dynamics:

- Conventional oil proved reserves
- Conventional oil reserve additions
- Conventional oil new discoveries
- Arctic oil
- Mined shale oil
- In-situ shale oil
- Light tight oil
- Mined oil sands
- In-situ oil sands (ultra-heavy oil)

The representation of uncertainty in TIAM-UCL for oil availability and costs differs between conventional and unconventional oil. For conventional oil, adapted country-level estimates of reserve and/or resource availability were taken from the literature and input into probability distributions, with corresponding assumptions on correlation between the estimates. For unconventional oil (e.g. mined bitumen), two parameters were assigned probability distributions: a range of estimates for original oil in-place (OOIP) and a range of estimates of a recovery factor (i.e. between 0 and 1, which determines the proportion of the in-place resource base which is technically recoverable). These two distributions were then combined using random repeated sampling (Monte Carlo simulations) to form regional estimates. The combination is the product of the OOIP and the

recovery factor, repeated a large number of times to generate an aggregated distribution. These estimates of the resource base for each category of oil were then combined with cost depletion curves, mostly formed from IEA data on cost ranges, and used to generate supply cost curves. For reference, a detailed discussion around the construction of cost depletion and supply cost curves taking into account the inherent uncertainty in volumetric and cost estimates across different regions and oil resource categories can be found in McGlade[43]. In general, the depletion analysis for unconventional oil exhibits significantly more rapid cost escalation (compared to conventional oil) as the resource base is depleted.

The mining processes for oil and fossil methane gas match the geological categories listed previously. Unconventional oil (tar sands and oil shale) has several more steps in the model to reflect the upgrading required to generate a barrel of crude oil (i.e. to get from bitumen/kerogen, to a barrel of synthetic crude oil). SI Table 15 shows the range of costs in TIAM-UCL for the mining technologies in the upstream sector. Also included is the region in TIAM-UCL containing the minimum and maximum cost for each category. As with fossil methane gas, the supply cost curves for each category of oil are split into three sections: the first 50% of the resource base considered the lowest cost, then the next 30%, and finally the most expensive oil representing the last 20% of the resource base.

SI Table 15. Cost ranges for oil resources in TIAM-UCL

Resource category	Minimum cost, $/boe	Minimum cost region	Maximum cost, $/boe	Maximum cost region
Proved reserves	11	MEA_OPEC	47	CSA_N
Reserve addition	21	MEA_OPEC	68	CSA_N
New discoveries	16	MEA_OPEC	94	IND
Light tight oil	30	USA	68	Outside North America
Arctic	46	-	97	-
Bitumen (mining)	51	Canada	89	Central and South America
Bitumen (in-situ)	43	Canada	88	Central and South America
Ultra-heavy oil	43	Central and South America	75	Central and South America
Oil shale	54	Europe	124	Middle East

* Sourced from McGlade, 2013[43] with light tight oil updated by Welsby, 2022[44]

Due to limited development outside of certain countries (e.g. Canada for bitumen production), costs have largely been applied homogenously across the relevant TIAM regions. SI Figure 23 shows the global supply cost curve for oil in TIAM-UCL split by region (a) and resource category (b). It should also be noted that unconventional oil is split into three separate cost categories: variable O&M, fixed O&M, and an investment cost (i.e. capital cost). In order to incorporate these into a supply cost curve with conventional oil, a singular supply cost figure was required, therefore the O&M costs were summed, and then a per-unit investment cost was assigned to each category of unconventional oil (derived by dividing cumulative investment and cumulative production from each mining technology) which then yielded a supply cost figure.

a)

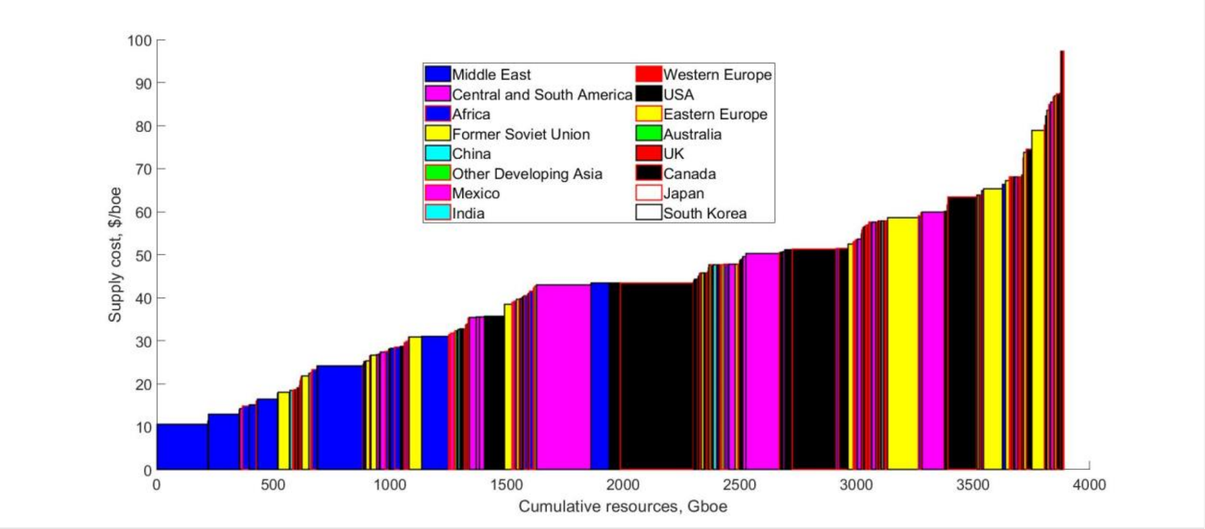

b)

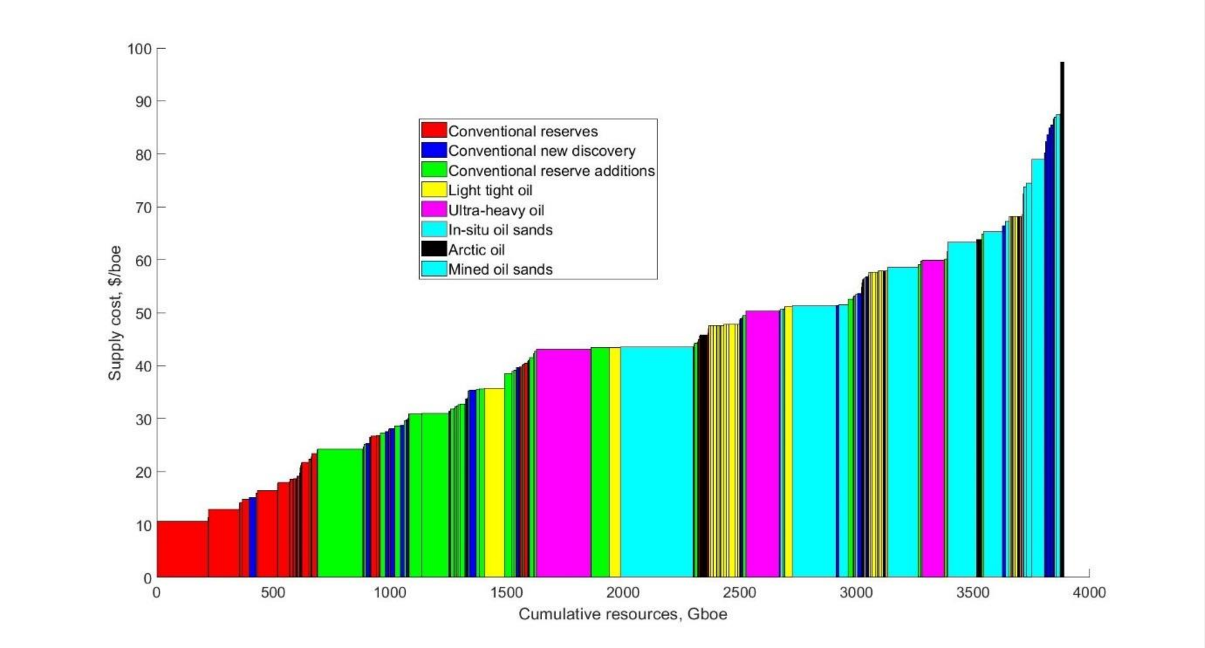

SI Figure 23. Global supply cost curve for oil from 2015. a) disaggregates supply curve by region and b) by resource category. Republished from Welsby, D., Price, J., Pye, S. & Ekins, P. Unextractable fossil fuels in a 1.5 °C world. Nature 597, 230–234 (2021)42 under Copyright © 2021, The Author(s), under exclusive licence to Springer Nature Limited.

Key upstream constraints on fossil fuel production

Coal

Upstream constraints for coal extraction are not as widely applied for two main reasons. Firstly, the extraction of coal does not follow the same geological production profile of oil and gas extraction; i.e. the growth and decline of production profiles through time and different geological structures. Secondly, for decarbonisation scenarios meeting 2°C and below, coal is rapidly phased out of the energy mix. Traditionally, this decline has not been constrained in previous iterations of TIAM-UCL, however in this paper we have constrained the rate at which coal can be phased out of the energy system.

Oil and fossil methane gas

There are a range of upstream user constraints which control the rate at which production of different categories of oil and gas can grow/decline. In short, these user constraints model the natural growth and decline of oil and fossil methane gas. The predominant form of constraint is an exponential (constant) rate of growth/decline across a time-slice, using seed values if there is no residual (historical) productive capacity. Equation 1 shows the functional form of these growth (a) and decline (b) constraints, for extractive technologies with historical production, while (c) and (d) shows the same equations for technologies which have no historical production and therefore require a seed value.

$$Production_{i,t} \leq (Production_{i,t-1} * Growth^{ts}) + Seed_{i,t} \quad (1\ (a))$$

$$Production_{i,t} \geq (Production_{i,t-1} * Decline^{ts}) + Seed_{i,t} \quad (1\ (b))$$

$$Production_{i,t} \leq Seed_{i,t} * Growth^{ts} \quad (1\ (c))$$

$$Production_{i,t} \geq Seed_{i,t} * Decline^{ts} \quad (1\ (d))$$

Where,

$Production_i$ = production of oil/gas for mining process i

$Seed_i$ = seed value from which growth/decline coefficients are assigned to if no historical (i.e. *t-1*) volumes, and which is added to overall growth/decline constraint across each time-slice

t = time period in the model (therefore *t-1* is the previous time-slice)

Growth = growth coefficient, where *Growth* ≥ 1

Decline = decline coefficient, where *Decline* ≤ 1

ts = time-slice length (i.e. *t – (t-1)*)

Therefore, for growth constraints, the production of an oil and/or gas mining technology in time slice *t* will be bounded (upper) by a maximum of production in time slice *t-1* multiplied by the growth coefficient to the power of the length of the time-slice. For decline constraints, the inverse applies: production in *t* will be bounded (lower) by a minimum of production in *t-1* multiplied by the decline coefficient to the power of the time-slice length. SI Table *16* shows examples of the growth/decline coefficient parameters and seed values used in TIAM-UCL, for a range of oil (a) and gas (b) mining technologies.

Additional constraints have been input as a proxy for controlling the expansion of associated fossil methane gas. Whilst the production itself is a function of oil extraction (and oil economics), the infrastructural issues surrounding associated gas utilisation require some degree of user constraint. Therefore, an upstream constraint is placed on the speed at which associated gas processing and separation capacity can be added.

SI Table 16. a) User constraints for a range of oil mining technologies extraction processes and b) gas extraction processes in TIAM-UCL

a)

Mining technology	Growth coefficient	Decline coefficient
Conventional proved reserves	1.41 ($\sqrt{2}$)	0.93
Conventional reserve additions	1.41 ($\sqrt{2}$)	0.93
Conventional undiscovered	1.41 ($\sqrt{2}$)	0.93
Shale oil	1.07	0.8
Mined bitumen	1.07	0.8
In-situ bitumen	1.1	0.85

b)

Mining technology	Growth coefficient	Decline coefficient
Conventional proved reserves	1.41 ($\sqrt{2}$)	0.95
Conventional reserve additions	1.41 ($\sqrt{2}$)	0.92
Conventional undiscovered	1.41 ($\sqrt{2}$)	0.92
Shale gas	1.27	0.83
Tight gas	1.12	0.83
Coal bed methane	1.12	0.83

Note: Regional variations are taken into account; therefore the numbers above may differ between regions. Additionally, the decline rate for conventional oil and gas fields will vary depending on the size of the field, the stage of decline, and the geological structure of the reservoirs. For example, larger fields generally exhibit slower rates of production decline, as shown with the value for conventional proved gas reserves which is taken from a representative decline parameter calculated from super-giant gas fields in the Former Soviet Union (e.g. Urengoy)[44].

Supply chain emissions from upstream fossil fuel activity

TIAM-UCL accounts for methane leakage from the gas supply chain, with a user-defined percentage of total fossil methane gas supply being lost into the atmosphere (i.e. direct methane emissions). Distinctions are made between conventional and unconventional fossil methane gas. A central methane leakage rate was derived across a range of literature[44]. The global warming potential of methane in TIAM-UCL is calculated over a 100-year time horizon (GWP-100), therefore where studies reported a different GWP time-period (e.g. 25 years), these were converted to GWP-100. For this study, our global average central leakage rate assumptions were 1.7% for conventional fossil methane gas and 2.1% for unconventional gas.

In addition to gas supply chains, TIAM-UCL also tracks methane emissions from oil and coal supply chains. For oil, fugitive methane emissions or intentional venting of methane is directly related to the presence of associated fossil methane gas. In short, if there is no infrastructure in place and/or demand downstream to absorb associated gas, the methane is either directly released into the atmosphere (venting) or flared (burnt-off) and released as CO_2. TIAM-UCL has various options for mitigating methane emissions from oil supply chains. Firstly, the model can build capacity to utilise rather than flare/vent fossil methane gas produced as a by-product of oil production.

Additionally, there is also a dummy option where the model can chose to flare rather than vent the methane if there is no demand for gas downstream/if building capacity is not cost optimal. Therefore, instead of methane being released into the atmosphere, CO_2 is instead emitted. As with

oil, there are options available to minimise methane emissions from coal mining. These include an option to gather the methane and inject it into pipelines for use downstream, or a dummy option to flare the gas, therefore releasing CO_2 into the atmosphere rather than methane.

Energy trade

Once extracted and processed, fossil fuels can then be transported between regions. An underlying trade matrix is used to determine inter-regional trade flow opportunities. For flexible forms of transportation (i.e. by maritime transport), the number of trade links will be higher than more constrictive forms of trading energy commodities (e.g. by pipeline, which are not just restricted by cost but also by geopolitical and geographical constraints).

Coal

It is assumed in TIAM-UCL that only higher-grade coal is traded; i.e. sub-bituminous, bituminous and anthracite. All trade flows for coal have been recalibrated in the model to ensure that 2015-2020 flows of coal around the world are consistent with historical data[45,46]. As with fossil methane gas (and oil) discussed subsequently, the trade of coal incurs costs, namely for its transportation via international shipping or across land-borders (i.e. by rail). The transportation costs, as with fossil methane gas and oil, are determined based on average shipping/train capacities and rental rates, and the distance between the regions. However, unlike fossil methane gas which requires processing, transformation and transportation infrastructure (e.g. liquefaction plants and pipelines), coal can be more easily transported and therefore it is assumed no investment costs are required.

Fossil methane gas

Fossil methane gas trade in TIAM-UCL is split between pipeline gas and liquefied natural gas (LNG). Both are constrained firstly by the underlying trade matrix. Additionally, trade volumes and infrastructure have been calibrated to 2015/2020-2025, with under construction infrastructure (both pipeline and LNG) fixed to come online in the model by 2020/2025, depending on an estimated start-date[47,48]. For example, SI Figure 24 shows under construction regasification capacity for China between 2016 and 2022, which is used to bound the build rates of trade infrastructure capacity.

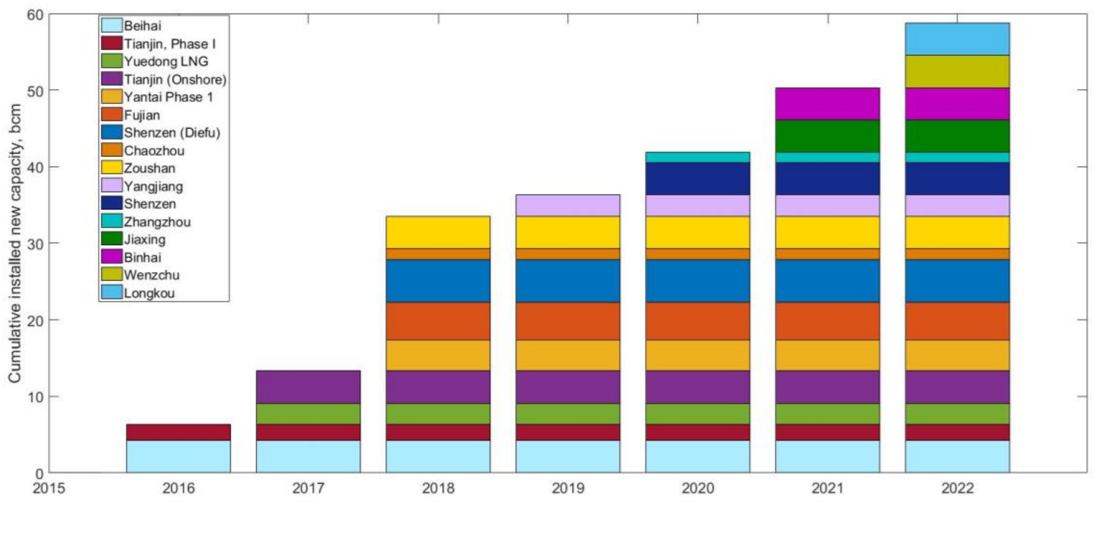

SI Figure 24. Cumulative installed regasification capacity in China, 2016-2022. Specific projects are denoted by the figure legend. Source: IGU, 2019[49]

Liquefied fossil methane gas trade in TIAM-UCL includes infrastructural parameters (liquefaction and regasification capacities, and build constraints) and cost parameters (CAPEX on new infrastructure, OPEX on the liquefaction/regasification process, and a shipping cost). Regionalised liquefaction costs have been included based on:

- Representative projects in each region, including the location of the liquefaction terminal and investment costs;
- Competition for E&P in recent years which led to real price inflation on projects built between 2010 and 2020[50];
- Whether the project was a brownfield extension or conversion (e.g. conversion of regasification (import) terminals in the United States into liquefaction (export) facilities), or green-field integrated project (e.g. Yamal LNG field and export facility development in Russia, and several projects in Australia including Gorgon, Ichthys (floating), and Wheatstone).

SI Table 17 shows a range of investment costs for liquefaction terminals in TIAM-UCL, showing the cost inflation attributed to a large range of projects coming online at the same time, and the corresponding stabilisation of these costs. It clearly shows which regions have the potential to take advantage of cost de-escalation for brownfield conversions/expansions[51], i.e. the USA and the Middle East, before (at least in this example) costs converge across regions for green-field investments. Additionally, the amount of capacity which can be converted / expanded under these lower costs has been limited to existing regasification capacity and/or a maximum upper limit based on proposed brownfield extensions.

SI Table 17. Liquefaction investment costs by region and year in TIAM-UCL, $M/PJ

Year	AFR	AUS	CAN	CHI	CSA	EEU	FSU	IND	JPN	MEA	MEX	ODA	SKO	UK	USA	WEU
2006	6	6	6	6	6	6	6	6	6	6	6	6	6	6	6	6
2010	16	16	16	14	16	18	16	14	16	9	16	14	14	18	9	18
2015	10	20	16	14	16	18	16	14	16	9	16	14	14	18	9	18
2020	10	21	22	20	20	20	20	20	20	10	20	20	20	20	12	20
2025	25	21	22	20	20	20	20	20	20	10	20	20	20	20	12	20
2050	20	21	20	20	20	20	20	20	20	18	20	20	20	20	12	20

* Data sourced from Welsby, 2022[43]. Note $M refers to million USD per petajoule.

LNG variable O&M (i.e. shipping) costs in TIAM-UCL are calculated based on a range of parameters[44,52]:

- Assumed distance between ports
- Average speed of tanker
- Average capacity of tanker; calculated based on average capacity of tankers which are assigned to fixed routes and/or average size of delivery
- Daily rental rate of tanker which is highly volatile depending on available capacities in each basin and seasonal spikes in LNG demand[53]; however, for a long-term energy systems model a fixed figure is assumed based on McGlade et al.[52].

- Boil-off rate (i.e. efficiency of transportation process translated into losses of fossil methane gas), which in turn is a function of journey time
- Loading/unloading time at each port

A database of LNG transportation costs has been developed[44], with representative average shipping costs between the TIAM-UCL regions used if more than one trade route is used. An example of these shipping costs between individual liquefaction and regasification terminals is shown in SI Table 18 below. For reference, the exporters are in red, and the zeros reflect that a) there is no intra-regional trade in TIAM-UCL and b) some regions are exogenously determined (through the trade link matrix shown) not to be able to trade with each other.

User constraints for fossil methane gas trade through LNG are employed for both the technology which covers overall export capacity (i.e. the liquefaction process technology) and the bilateral trade process itself. In short, this constrains the model from building new capacity too quickly and sending all of the potential output through a single trade link.

SI Table 18. Representative shipping costs for LNG between TIAM-UCL regions, $M/PJ

	**AFR**	**AUS**	**CAN**	**CHI**	**CSA**	**EEU**	**FSU**	**IND**	**JPN**	**MEA**	**MEX**	**ODA**	**SKO**	**UK**	**USA**	**WEU**
AFR	0	0	0	1.09	0.26	0	0	0.84	1.28	0.63	1.06	1.02	1.19	0.83	0	0.81
AUS	0	0	0	0.75	0	0	0	0.96	0.8	0	0	0.73	0.8	0	0	0
CAN	0	0	0	0.85	0	0	0	1.2	0.81	0	0.62	0.85	0.82	0	0	0
CHI	0	0	0	0	0	0	0	0	0	0	0	0	0	0	0	0
CSA	0	0	0	1.07	0	0	0	0.97	1.14	0.91	0.78	1.15	1.12	0.81	0	0.8
EEU	0	0	0	0	0	0	0	0	0	0	0	0	0	0	0	0
FSU	0	0	0	0.72	0	0	0	1.39	0.59	0	0	1.38	0.66	0.81	0	0.8
IND	0	0	0	0	0	0	0	0	0	0	0	0	0	0	0	0
JPN	0	0	0	0	0	0	0	0	0	0	0	0	0	0	0	0
MEA	0	0	0	0.83	0.81	1.02	0	0.6	0.94	0	1.28	0.74	0.92	0.97	0	0.85
MEX	0	0	0	0	0	0	0	0	0	0	0	0	0	0	0	0
ODA	0	0	0	0.66	0	0	0	0.71	0.73	0	0	0	0.69	0	0	0
SKO	0	0	0	0	0	0	0	0	0	0	0	0	0	0	0	0
UK	0	0	0	0	0	0	0	0	0	0	0	0	0	0	0	0
USA	0	0	0	1.09	0.84	0.91	0	1.21	1.11	0	0.72	1.1	1.09	0.85	0	0.9
WEU	0	0	0	0	0	0.63	0.64	0	0	0	0	0	0	0	0	0

* Data sourced from Welsby, 2022[44]

For pipeline investment costs and capacity additions in the near-term, individual project costs and capacity have been added where appropriate (e.g. pipeline cost and maximum volume from Russia to China between 2015 and 2020 are based on the Power of Siberia pipeline, which came online at the end of 2019). Some examples of pipeline investment costs are shown in SI Table 19, with each pipeline at different development stage[44]. However, other factors need to be taken into account including whether the pipeline has to cross challenging physical barriers (e.g. a sea or mountainous territory).

SI Table 19. Pipeline investment costs for a range of representative projects used in TIAM-UCL

Pipeline Name	Status	Investment Cost, $M/PJ	Investment Cost, $M/km
Power of Siberia	Operational	10.38-23.02	5.27-11.67
Central Asia-China	Operational	3.51	3.99
TAPI	Proposed	6.66-8.33	7.11-8.89

Additionally, a user constraint has been added as an upper bound on potential gas pipeline trade, with a similar functional form as the upstream constraints discussed in the section that follows. In short, it is assumed that the model can, at a maximum, double capacity across a ten year period for any trade route (e.g. add a new pipeline parallel to an existing one with the same capacity[44]. Therefore, an exponential growth constraint is set in the following form shown in Equation 2:

$$PipeCap_{a \to b,t} \leq PipeCap_{a \to b,t-1} * PipeGro^{ts} + Seed_{r,t} \tag{2}$$

Where,

$PipeCap_{a \to b,t}$ = pipeline capacity between exporter *a* and importer *b*, in time period *t*

$PipeCap_{a \to b,t-1}$ = pipeline capacity between exporter *a* and importer *b*, in time period *t-1*, i.e. the preceding time period

$PipeGro^{ts}$ = pipeline growth coefficient, set at ~1.07 (i.e. allows a doubling of capacity over 10 years using the above formulation)

$Seed_{r,t}$ = seed value for region *r* and time-period *t*, which allows growth value to take hold if there is no historical trade link, or adds on to the growth constraint for absolute upper bound (i.e. slackness on the constraint). The seed value is added across the time-slice, rather than in each individual year. As with LNG, this is based on a maximum capacity addition across a time-slice.

For regions where volumes of trade are already well established and there is significant pipeline capacity in place, the seed value has been set to zero from 2020 (e.g. between the UK and Western Europe, and the USA and Canada). A seed value is included in these cases between 2006 and 2015 in case large increases in gas pipeline trade were in evidence, such as between the United States and Mexico after the expansion of shale gas in the Barnett shale play. In short, the seed value allows the model to expand trade up to the upper bounds which have been added for 2015 to calibrate fossil methane gas trade to historical data. However, some trade links have a seed value from 2020 to allow the model to expand pipeline capacity over the growth coefficient alone. For example, the seed value for gas pipeline trade between the Former Soviet Union and China is bounded (upper) by the growth coefficient (1.07/a) and a seed value equivalent to the Power of Siberia pipeline operating between a minimum (70%) and maximum (90%) contracted quantity[44].

Oil

The trade of oil commodities is split into various different products, which are outputs of processing / transformation processes in the upstream: crude oil, heavy fuel oil, naphtha, gas liquids, and diesel. As with fossil methane gas trade via LNG tankers, the variable cost of transporting oil via tankers is assumed to be a function of the distance between ports, the speed of the tanker, and the average capacity of a ship travelling from the exporter to the importer.

SI Section 6. Detailed representation of Supply curves for Russia and Saudi Arabia

As part of this scenario analysis, TIAM-UCL was further modified to allow us to explore the impact of geopolitical uncertainties on the energy system transition across different climate futures. All recent updates to TIAM-UCL from recent papers[33,54] have been incorporated into this model version.

Supply cost curves

To represent geopolitical scenarios in TIAM-UCL, we have focused on disaggregating the upstream sector of the model. In particular, we have disaggregated Russia and Saudi Arabia from their respective regions. In order to do this, we have split out the Former Soviet Union and Middle East oil and natural gas supply cost curves to allow us to explicitly represent production from Russia and Saudi Arabia.

Numerous sources were used in the first instance to break apart these supply cost curves including BP[55], IEA[47,56], McGlade[43], Rystad[57] and Welsby[44]. SI Figure 10 below shows the use of disaggregated field level data from Welsby to explicitly represent supply cost curve characteristics for Russian non-associated natural gas when taken independently from the rest of the Former Soviet Union region. By doing this, we can construct scenarios which explicitly constrain the production of Russian natural gas, including the respective flow of gas to pipeline and LNG export facilities.

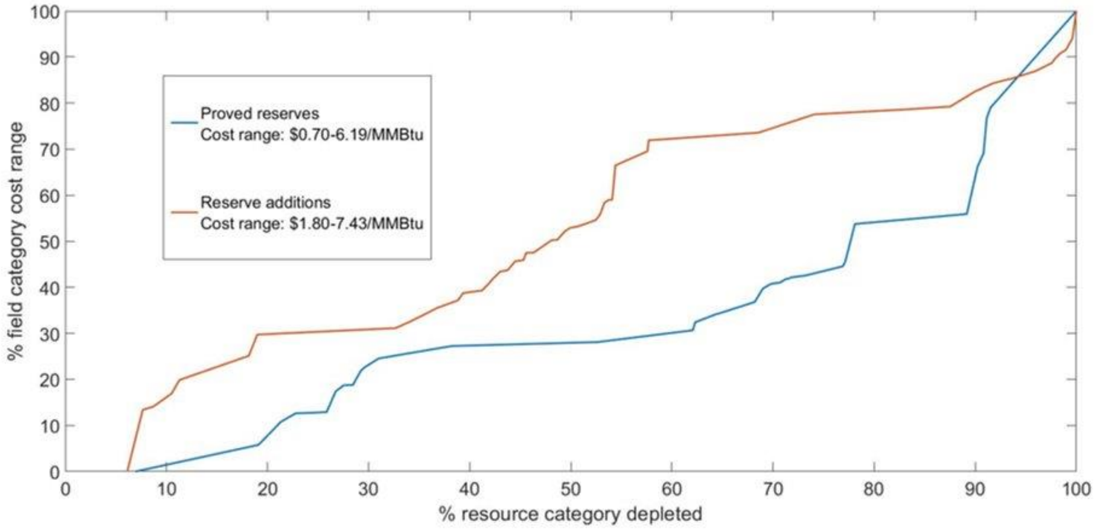

SI Figure 25. Cost depletion curves for Russian non-associated natural gas proved reserves and reserve additions. Depletion curve measures the increase in costs as resources are depleted. Republished from Welsby, D. Modelling uncertainty in global gas resources and markets. (University College London, London, 2022)[44], and released under a Creative Commons Attribution-Non Commercial 4.0 International Licence.

Additionally, SI Table 20 below shows the new supply cost steps for Russia and other FSU countries (i.e. Central Asia and the Caspian) for proved reserves of non-associated natural gas.

SI Table 20. Supply cost steps for proved non-associated natural gas reserves for Russian and other countries in the Former Soviet Union region

Country/region	Proved reserves cost step	Proved reserve volume, tcm	Cost, $/GJ
Russia	1	15.6	1.53
	2	9.4	2.81
	3	6.2	4.74
Rest of Former Soviet Union	1	5.4	2.72
	2	3.3	3.91
	3	2.2	5.30
Previous FSU region	1	21	1.66
	2	12.7	3.48
	3	8.4	5.79

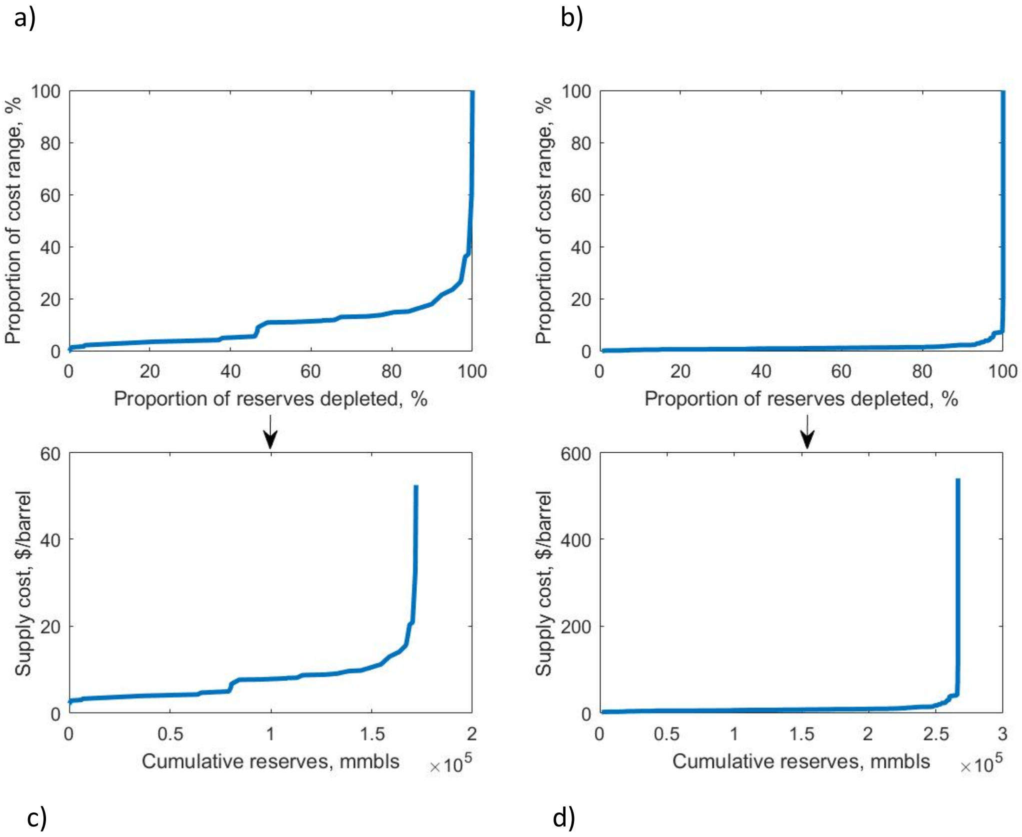

SI Figure 26. Cost depletion and supply cost curves for Saudi Arabia (a, c) and the rest of Middle Eastern OPEC countries (b, d). Data sourced from Rystad U-Cube database and Welsby (2022)[44,57]

SI Table 21 shows the new supply cost steps for Saudi Arabia and other MEA OPEC countries for proved reserves of crude oil.

SI Table 21. Supply cost steps for proved crude oil reserves for Saudi Arabia and other countries in the Middle East OPEC region

Country/region	Proved reserves cost step	Proved reserve volume, Gb	Cost, $/boe ($/GJ)
Saudi Arabia	1	86.0	9.40 (1.65)
	2	51.6	11.98 (2.10)
	3	34.4	16.53 (2.90)
Rest of Middle East OPEC countries	1	133.2	10.55 (1.85)
	2	79.9	12.85 (2.25)
	3	53.3	27.53 (4.83)
Previous MEA-OPEC region	1	219.2	10.55 (1.85)
	2	131.5	12.85 (2.25)
	3	87.7	16.36 (2.87)

Dynamic growth constraints

In order to model growth and decline dynamics of oil and gas production, additional asymmetric user constraints were added for the new mining technologies for Saudi Arabia (oil) and Russia (oil and non-associated natural gas). These are the same as pre-existing constraints for the aggregated regions in TIAM-UCL.

Trade in TIAM-UCL

To implement different geopolitical scenarios in TIAM-UCL, we have also disaggregated the trade moduleso oil and gas exports from Saudi Arabia and Russia are separate from the rest of the aggregated Former Soviet Union and Middle Eastern regions. We can therefore explicitly model scenarios based on the trade of fossil fuels from Russia and Saudi Arabia, rather than relying on proxy constraints for the wider respective regions.

To do this we have disaggregated and recalibrated the trade module in TIAM-UCL:

- International trade flows (i.e. inter-regional) between 2005 and 2020 from Russia and other FSU countries
- Pipeline capacities (for natural gas) from Russia and other Former Soviet Union countries built since 2005 using data from Welsby[44]:
  - Russian pipelines to Turkey and Europe - Nord Stream 1, TurkStream
  - Azerbaijan pipelines to Turkey – Southern Gas Corridor
  - Turkmen (including Kazakhstan and Uzbekistan) pipeline to China – Central Asia gas pipeline
  - Russia pipeline to China – Power of Siberia
- Under construction pipelines:
  - Southern Gas Corridor
- Pipeline investment costs
- International trade flows (i.e. inter-regional) between 2005 and 2020 from Saudi Arabia and other MEA countries

Oil trade is less reliant on specific infrastructural investments. In 2015, over 60% of total global oil production was traded via maritime routes (i.e. in oil tankers)[58], and therefore oil has been seen as a global market given this flexibility. In terms of traded volumes, this means that shipping accounts for over 90% of global oil trade[58]. Whilst gas markets are becoming increasingly integrated, the infrastructural requirements and the continued presence of indexed long-term contracts means that gas markets, as of yet, do not exhibit the same uniform global market.

References

1. Watson, J. China's Hidden Dragon: bringing gas from west to east. *Energy Eonomists* (2002).
2. Li, X. *Natural Gas in China: A Regional Analysis*. https://www.oxfordenergy.org/wpcms/wp-content/uploads/2015/11/NG-103.pdf (2015).
3. O'Sullivan, S. *China's Natural Gas Development Report: A Tale of Two Years*. https://www.oxfordenergy.org/wpcms/wp-content/uploads/2021/02/Insight-85-Chinas-Natural-Gas-Development-Report.pdf (2021).
4. UNFCCC. China's Achievements, New Goals and New Measures for Nationally Determined Contributions. Preprint at https://unfccc.int/sites/default/files/NDC/2022-06/China's Achievements%2C New Goals and New Measures for Nationally Determined Contributions.pdf (2020).
5. IEA. Natural gas markets remain tight as uncertainty persists around Chinese LNG demand and further supply cuts by Russia - News. Preprint at https://www.iea.org/news/natural-gas-markets-remain-tight-as-uncertainty-persists-around-chinese-lng-demand-and-further-supply-cuts-by-russia (2023).
6. IEA. Coal Market Update – July 2023 – Analysis. *IEA* Preprint at https://www.iea.org/reports/coal-market-update-july-2023 (2023).
7. Meidan, M. Chinas climate and energy policy after the Two Sessions. Preprint at https://a9w7k6q9.stackpathcdn.com/wpcms/wp-content/uploads/2023/04/Chinas-climate-and-energy-policy-after-the-Two-Sessions.pdf (2023).
8. Zhang, J., Meerman, H., Benders, R. & Faaij, A. Potential role of natural gas infrastructure in China to supply low-carbon gases during 2020–2050. *Appl Energy* **306**, 117989 (2022).
9. Ji, Q., Fan, Y., Troilo, M., Ripple, R. D. & Feng, and L. China's Natural Gas Demand Projections and Supply Capacity Analysis in 2030. *The Energy Journal* **Volume 39**, (2018).
10. Li, J. *et al.* Natural gas industry in China: Development situation and prospect. (2020) doi:10.1016/j.ngib.2020.04.003.
11. National Energy Administration. *China Natural Gas Development Report*. http://www.nea.gov.cn/1310139334_16294604014501n.pdf (2021).
12. Xu, G., Chen, Y., Yang, M., Li, S. & Marma, K. J. S. An outlook analysis on China's natural gas consumption forecast by 2035: Applying a seasonal forecasting method. *Energy* **284**, 128602 (2023).
13. Pan, X. *et al.* Implications of near-term mitigation on China's long-term energy transitions for aligning with the Paris goals. *Energy Econ* **90**, 104865 (2020).
14. Ma, X., Peng, T., Zhang, Y., Wang, L. & Pan, X. Accelerating carbon neutrality could help China's energy system align with below 1.5 °C. *J Environ Manage* **337**, 117753 (2023).
15. Pan, X. *et al.* Analysis of China's oil and gas consumption under different scenarios toward 2050: An integrated modeling. *Energy* **195**, 116991 (2020).

16. SENR-RMU. Research on China's carbon emissions pathway under the 1.5℃ target 1.5℃温升目标下中国碳排放路径研究. Preprint at http://www.climatechange.cn/CN/10.12006/j.issn.1673-1719.2020.228 (2021).

17. ICCSD. *China's Long-Term Low-Carbon Development Strategies and Pathways: Comprehensive Report*. (Springer Singapore, Singapore, 2022).

18. CNPC. Unpacking CNPC's net-zero road map for China | S&P Global Commodity Insights. Preprint at https://www.spglobal.com/commodityinsights/en/market-insights/blogs/energy-transition/020822-china-net-zero-road-map-cnpc (2022).

19. Tracker, C. A. 2100 Emissions and expected warming based on pledges and current policies. November 2022. Available at: https://climateactiontracker.org/global/temperatures/. Copyright ©2022 by Climate Analytics and NewClimate Institute. All rights reserved. Preprint at (2022).

20. Geiges, A. *et al.* Incremental improvements of 2030 targets insufficient to achieve the Paris Agreement goals. *Earth System Dynamics* **11**, 697–708 (2020).

21. Chen, J.-W., Cui, H.-J., Chen, N. & Qian, G.-Q. Uncertainty of temperature rise under nationally determined contributions and carbon neutral policies. *Advances in Climate Change Research* **14**, 580–586 (2023).

22. IPCC. Working Group III Contribution to the Sixth Assessment Report of the Intergovernmental Panel on Climate Change. (2023).

23. Equinor. Intergated annual report 2022. Preprint at https://cdn.equinor.com/files/h61q9gi9/global/03d92ebc1ab4f124aabe4fa5be40da3dec6e24b4.pdf?2022-annual-report-equinor.pdf (2023).

24. IEA. World Energy Outlook 2023 – Analysis. *IEA* Preprint at https://www.iea.org/reports/world-energy-outlook-2023 (2023).

25. IEEJ. IEEJ Outlook 2023 Energy, Environment and Economy Challenges for achieving both energy security and carbon neutrality. Preprint at https://eneken.ieej.or.jp/data/10974.pdf (2023).

26. BP. *BP Energy Outlook 2022*. http://files/872/bp Energy Outlook 2022.pdf.

27. OPEC. OPEC : World Oil Outlook. Preprint at https://www.opec.org/opec_web/en/publications/340.htm (2022).

28. Shell. Energy security scenarios full report.pdf. Preprint at https://www.shell.com/energy-and-innovation/the-energy-future/scenarios/the-energy-security-scenarios/_jcr_content/root/main/section_926760145/simple/promo/links/item0.stream/1679344984968/5bc8327925d66e1402040d0e79fed7291bf9b7e9/energy-security-scenarios (2023).

29. Riahi, K. *et al.* The Shared Socioeconomic Pathways and their energy, land use, and greenhouse gas emissions implications: An overview. *Global Environmental Change* **42**, 153–168 (2017).

30. Daioglou, V., Stehfest, E., Wicke, B., Faaij, A. & van Vuuren, D. P. Projections of the availability and cost of residues from agriculture and forestry. *GCB Bioenergy* **8**, 456–470 (2016).

31. Ricardo Energy & Environment. Biomass Feedstock Availability, Final Report for BEIS. *Ref: ED662421043 - Issue Number 3* (2017).

32. Hoogwijk, M., Faaij, A., de Vries, B. & Turkenburg, W. Exploration of regional and global cost-supply curves of biomass energy from short-rotation crops at abandoned cropland and rest land under four IPCC SRES land-use scenarios. *Biomass Bioenergy* **33**, 26–43 (2009).

33. Muttitt, G., Price, J., Pye, S. & Welsby, D. Socio-political feasibility of coal power phase-out and its role in mitigation pathways. *Nat Clim Chang* **13**, 140–147 (2023).

34. Dodds, P. & McDowall, W. *A Review of Hydrogen Production Technologies for Energy System Models*. (2012).

35. Ekins, P. *et al. The Role of CCS in Meeting Climate Policy Targets*. (2017).

36. Rubin, E. S., Davison, J. E. & Herzog, H. J. The cost of CO2 capture and storage. *International Journal of Greenhouse Gas Control* **40**, 378–400 (2015).

37. IRENA International Renewable Energy Agency. *Renewable Power Generation Costs in 2017*. *International Renewable Energy Agency* (2018). doi:10.1007/SpringerReference_7300.

38. IEA PVPS. *Trends 2018 in Photovoltaic Applications*. *Survey Report of Selected IEA Countries between 1992 and 2014* (2018).

39. Butnar, I., Broad, O., Solano Rodriguez, B. & Dodds, P. E. The role of bioenergy for global deep decarbonization: CO2 removal or low-carbon energy? *GCB Bioenergy* **12**, 198–212 (2020).

40. Chen, C. & Tavoni, M. Direct air capture of CO2and climate stabilization: A model based assessment. *Clim Change* **118**, 59–72 (2013).

41. Remme, U., Blesl, M. & Fahl, U. *Global Resources and Energy Trade: An Overview for Coal, Natural Gas, Oil and Uranium*. (2007).

42. Welsby, D., Price, J., Pye, S. & Ekins, P. Unextractable fossil fuels in a 1.5 °C world. *Nature* **597**, 230–234 (2021).

43. McGlade, C. Uncertainties in the outlook for oil and gas. *Doctoral thesis, UCL (University College London).* (2013).

44. Welsby, D. Modelling uncertainty in global gas resources and markets. (University College London, London, 2022).

45. BP. *BP Statistical Review of World Energy*. (2019).

46. IEA. *Coal Information 2019*. (2019).

47. IEA. *Natural Gas Information 2019*. https://www.iea.org/reports/natural-gas-information-2019 (2019).

48. IGU. *World LNG Report 2019*. https://www.igu.org/sites/default/files/node-news_item-field_file/IGU Annual Report 2019_23 loresfinal.pdf (2019).

49. IGU. *Global Natural Gas Insights*. https://www.igu.org/global-natural-gas-insights (2019).

50. Songhurst, B. *LNG Plant Cost Escalation*. https://www.oxfordenergy.org/wpcms/wp-content/uploads/2014/02/NG-83.pdf?v=79cba1185463 (2014).

51. Songhurst, B. *LNG Plant Cost Reduction 2014–18*. https://www.oxfordenergy.org/wpcms/wp-content/uploads/2018/10/LNG-Plant-Cost-Reduction-2014–18-NG137.pdf?v=79cba1185463 (2018).

52. McGlade, C., Bradshaw, M., Anandarajah, G., Watson, J. & Ekins, P. *A Bridge to a Low-Carbon Future? Modelling the Long-Term Global Potential of Natural Gas*. https://ukerc.ac.uk/publications/gas-as-a-bridge/ (2014).

53. Hellenic Shipping News. Could LNG shipping spot rates hit $250,000/day? https://www.hellenicshippingnews.com/could-lng-shipping-spot-rates-hit-250000-day/ (2018).

54. Pye, S. *et al.* Regional uptake of direct reduction iron production using hydrogen under climate policy. *Energy and Climate Change* **3**, 100087 (2022).

55. BP. *BP Statistical Review of World Energy 2022*. https://www.bp.com/content/dam/bp/business-sites/en/global/corporate/pdfs/energy-economics/statistical-review/bp-stats-review-2022-full-report.pdf (2022).

56. IEA. *Oil Information 2019*. https://www.oecd-ilibrary.org/energy/oil-information-2019_553cdfcc-en (2019).

57. Rystad. Rystad 2021 UCube Browser. www.rystaden ergy.com/energy-themes/oil-gas/upstream/u-cube/ (2021).

58. EIA. *World Oil Transit Chokepoints*. https://www.eia.gov/international/content/analysis/special_topics/World_Oil_Transit_Chokepoints/wotc.pdf (2107).
