# Supplementary material for: The global implications of a Russian gas pivot to Asia
Source: Nat Commun. 2025 Jan 4;16:386. doi: 10.1038/s41467-024-55697-7 (PMC11700154; doi:10.1038/s41467-024-55697-7)
Supplement: Supplementary file 2 — Description of Additional Supplementary Files [file 41467_2024_55697_MOESM2_ESM.pdf]

Description of Additional Supplementary Files

File Name: Supplementary Data 1
Description: This file contains the data values used in the manuscript graph-based figures
